# Anthocyanin accumulation correlates with hormones in the fruit skin of ‘Red Delicious’ and its four generation bud sport mutants

**DOI:** 10.1186/s12870-018-1595-8

**Published:** 2018-12-18

**Authors:** Wen-Fang Li, Juan Mao, Shi-Jin Yang, Zhi-Gang Guo, Zong-Huan Ma, Mohammed Mujitaba Dawuda, Cun-Wu Zuo, Ming-Yu Chu, Bai-Hong Chen

**Affiliations:** 1 College of Horticulture, Gansu Agricultural University, Lanzhou, 730070 People’s Republic of China; 2 Department of Horticulture, FoA, University for Development Studies, 1882 Tamale, Ghana

**Keywords:** Apple, Bud sport, Fruit skin, Pigmentation, RNA-seq, Hormone

## Abstract

**Background:**

Bud sport mutants of apple (*Malus domestica* Borkh.) trees with a highly blushed colouring pattern are mainly caused by the accumulation of anthocyanins in the fruit skin. Hormones are important factors modulating anthocyanin accumulation. However, a good understanding of the interplay between hormones and anthocyanin synthesis in apples, especially in mutants at the molecular level, remains elusive. Here, physiological and comparative transcriptome approaches were used to reveal the molecular basis of color pigmentation in the skin of ‘Red Delicious’ (G0) and its mutants, including ‘Starking Red’ (G1), ‘Starkrimson’ (G2), ‘Campbell Redchief’ (G3) and ‘Vallee spur’ (G4).

**Results:**

Pigmentation in the skin gradually proliferated from G0 to G4. The anthocyanin content was higher in the mutants than in ‘Red Delicious’. The activation of early phenylpropanoid biosynthesis genes, including *ASP3*, *PAL*, *4CL*, *PER*, *CHS*, *CYP98A* and *F3’H*, was more responsible for anthocyanin accumulation in mutants at the color break stage. In addition, IAA and ABA had a positive regulatory effect on the synthesis of anthocyanins, while GA had the reverse effect. The down-regulation of *AACT1*, *HMGS*, *HMGR*, *MVK*, *MVD2*, *IDI1* and *FPPS2* involved in terpenoid biosynthesis influences anthocyanin accumulation by positively regulating transcripts of *AUX1* and *SAUR* that contribute to the synthesis of IAA, *GID2* to GA, *PP2C* and *SnRK2* to ABA. Furthermore, MYB and bHLH members, which are highly correlated (*r*=0.882–0.980) with anthocyanin content, modulated anthocyanin accumulation by regulating the transcription of structural genes, including *CHS* and *F3’H*, involved in the flavonoid biosynthesis pathway.

**Conclusions:**

The present comprehensive transcriptome analyses contribute to the understanding of the the relationship between hormones and anthocyanin synthesis as well as the molecular mechanism involved in apple skin pigmentation.

**Electronic supplementary material:**

The online version of this article (10.1186/s12870-018-1595-8) contains supplementary material, which is available to authorized users.

## Background

Somatic mutations which are also referred to as 'bud sports’ usually occur in woody plant species [1, 2]. and the genetic background of these mutants is nearly identical to that of their parents [3, 4]. However, epigenetic changes causing fruit colour alteration in apple have been identified (*Malus domestica* Brokh.) [2, 5]. Fruit skin colour is a key appearance and nutrition quality attribute of apple fruit [6]. Anthocyanins are among the secondary metabolites that contribute to the colours of fruits [7]. Pigmentation in the skin of apple fruit varies among cultivars and is influenced by environmental factors, including temperature [8] and the level of sunlight irradiation [9, 10]. Furthermore, hormones are likely to be important factors that modulate light-dependent anthocyanin accumulation [11–13]. In summary, exploring the molecular mechanisms of hormones and anthocyanin synthesis in apple fruit and its bud sport mutants is crucial to research on pigment accumulation and plant somatic mutation.

Previous studies have shown that auxin (IAA), cytokinin (CTK), gibberellins (GA), jasmonate acid (JA), abscisic acid (ABA) and ethylene (ETH) interact in controlling anthocyanin biosynthesis [11, 13–16]. In addition, the identification and functional characterization of MYB and bHLH transcriptional factors revealed that they play a role in structural gene transcription. These structural genes include chalcone isomerase (*CHI*), chalcone synthase (*CHS*), flavonol synthase (*FLS*), leucoanthocyanidin reductase (*LAR*), flavonoid 3’-hydroxylase (*F3’H*) and anthocyanidin reductase (*ANR*), which are involved in the anthocyanin biosynthesis pathway [17–21]. MYB proteins are characterized by two imperfect repeats of the DNA-binding motifs R2 and R3 [22], and bHLH proteins are characterized by the basic helix-loop-helix domain, which is responsible for sequence-specific DNA binding [23].

The publication of the apple reference genome [24] and the development of new tools for transcriptomics have facilitated recent advances in the genome-wide analysis of dynamic gene expression during fruit development [2, 25]. The strategies of hormone and anthocyanin synthesis are often applied without a full understanding of the effect at the molecular level, with the exception of a few studies that have correlated biochemical and physiological outcomes with transcriptomic changes [11–13]. Apple cultivar ‘Red Delicious’ (*M. domestica*) is the most frequently captured sport apple variety that is usually selected on the phenotypic basis of spur type and intense red fruit colour. The cultivar’s four continuous generation mutants, namely, ‘Starking Red’, ‘Starking Red’, ‘Starkrimson’, ‘Campbell Redchief’ and ‘Vallee Spur’, have been screened. Therefore, a comparative transcriptome analysis combined with physiological and biochemical characteristics were employed to investigate the relationship between hormone and anthocyanin synthesis on the accumulation of apple skin pigment.

## Materials and methods

### Plant material

**Fig. 1:**
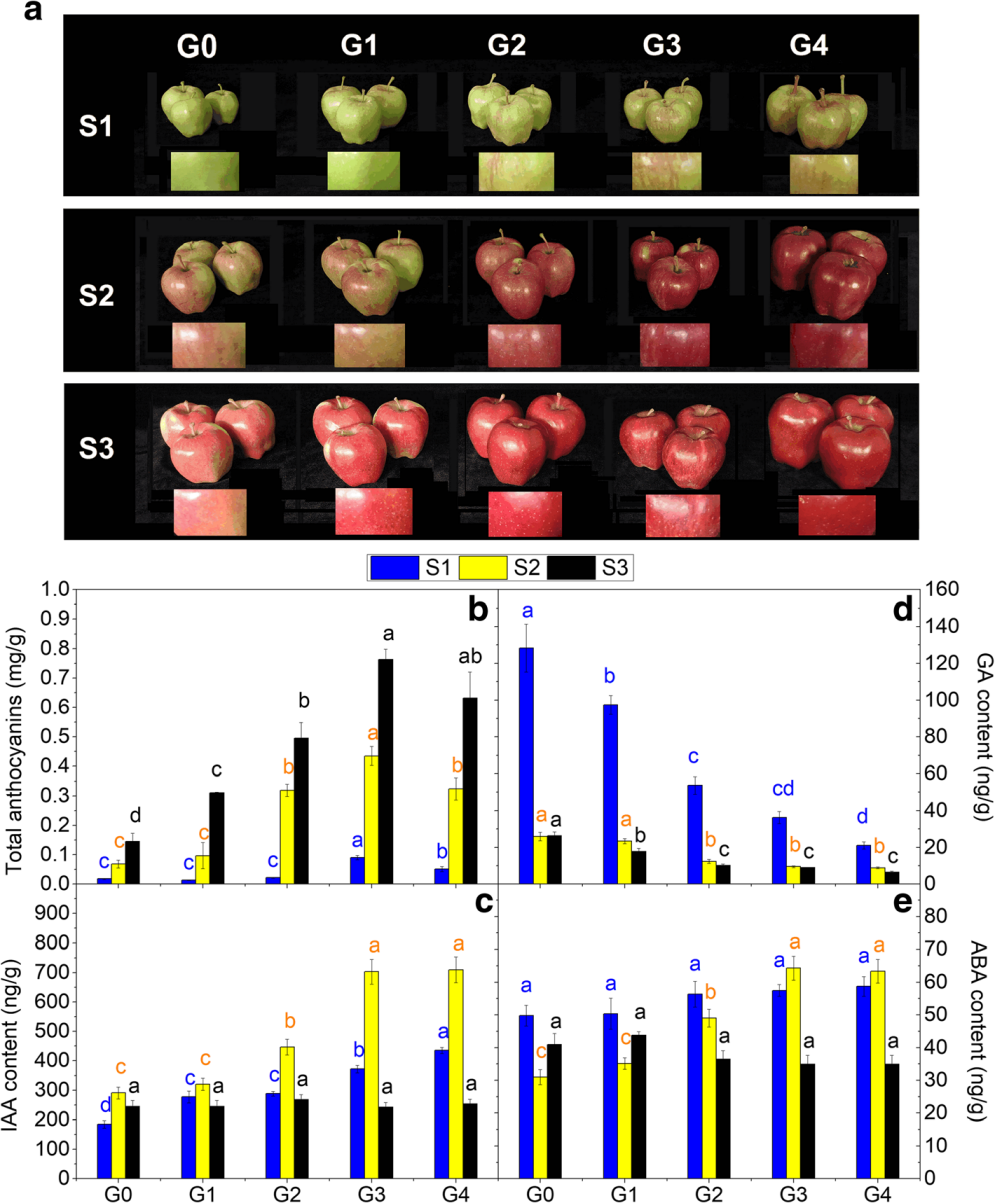
**a** Close-up views of ‘Red Delicious’ and its four generation mutants (‘Starking Red’, ‘Starkrimson’, ‘Campbell Redchief’ and ‘Vallee spur’), named G0 to G4, at three developmental stages (S1–S3) used for anthocyanin quantification, transcriptome profiling and qRT-PCR. **b** The changes in total anthocyanin concentrations in the fruit skin of the five strains at S1 to S3. Changes in endogenous hormone levels, including IAA (**c**), GA (**d**) and ABA (**e**), in the fruit skin of the five strains at S1 to S3. Values are means ± SE. Different lower case letters indicate significant differences among the five strains (*P*=0.05)

‘Red Delicious’ is the most frequently captured sport apple variety, featuring four continuous generation mutants. ‘Starking Red’ is a bud sport from ‘Red Delicious’ and a typical representative of the first generation. The second generation is ‘Starkrimson’, which is a bud sport from the first-generation ‘Starking Red’. The fourth generation, ‘Vallee Spur’, is a bud sport of the third generation, ‘Campbell Redchief’, which is bud sport of ‘Starkrimson’. Mature apple fruit of these five cultivars range from having red vertical stripes to being completely red (Fig. 1a).

Fruit samples of ‘Red Delicious’ and its four continuous generation mutants (‘Starking Red’, ‘Starkrimson’, ‘Campbell Redchief’ and ‘Vallee spur’) were named G0 to G4 and collected in 2017 from 12-year-old trees grown in apple demonstration gardens at Tianshui, China. Briefly, 20−30 fruits from each of the five strains were sampled at three developmental stages, i.e., 5 August (S1), 25 August (S2), and 14 September (S3) (Fig. 1a). Before color break, at color break and fruit maturity stages are designated by S1, S2 and S3, respectively. The entire skin samples were removed with 1 mm of the cortical tissue and collected from 6 fruits per replicate with three independent biological replicates. All samples were collected at the same time of day (9–10 _AM_), immediately frozen in liquid nitrogen and stored at −80 °C for further analysis of anthocyanin contents, endogenous hormone contents and gene expression profiles (qRT-PCR). In addition, samples from S2 were used for RNA-seq analysis.

### Anthocyanin quantification

Lyophilized apple skin samples were finely ground, and 500 mg of powdered samples was homogenized in 10 mL of methanol with 1% HCl. The homogenate was transferred to a calibration test tube with a constant volume of 20 mL and kept for 20 min at 4 °C with shaking under dark conditions. Then, the samples were filtered through a 0.2 μm polyethersulfone filter (Krackeler Scientific, Inc., Albany, NY, USA) and analysed using a TU-1900 double beam UV-visible spectrophotometer (Beijing Purkinje General Instrument Co. LTD). Anthocyanin levels were calculated by dividing the absorbance by the coefficient of regression (0.0214) acquired by standard scale measurements.

### Determination of IAA, GA and ABA concentrations

A total of 1.0 g of each lyophilized apple skin samples were used for phytohormone extractions [26]. The apparatus used for high-performance liquid chromatography (HPLC) was the LC-20AD system (Shimadzu, Kyoto, Japan) equipped with a Zorbax Eclipse Plus C18 column (4.6 mm × 250 mm × 5.0 μm, Agilent, Palo Alto, CA, USA) and an SPD-20A UV detector. The determination method was performed with different concentrations of IAA, GA, and ABA, standard samples, which were used to construct a standard curve [27].

### RNA extraction

Total RNA was extracted from approximately 200 mg of lyophilized apple skin samples ground in liquid nitrogen using the RNase-Free DNase Set (Qiagen, Valencia, CA, USA) and then cleaned with the RNeasy Mini Kit (Qiagen). RNA quality and quantity were determined using a Pultton P200 Micro Volume Spectrophotometer (Pultton Technology Limited).

### RNA-seq library preparation and construction

The 5 triplicate samples (5 varieties at S2) yielded 15 nondirectional cDNA libraries with a total of 68.18 million reads (Table 1), which were prepared from 3.0 μg of total RNA using the NEBNext, Ultra^TM^ RNA Library Prep Kit (NEB, USA). The process of library construction was described in detail by Mao et al. (2017) [26, 27], and the 15 libraries were sequenced on an Illumina HiSeq 2000 platform.

**Table 1:** Summary of RNA-Seq data and mapping metrics

Variety	Replicate	Total reads	Clean reads	Mapped reads	Average reads	Average mapped reads	Quality (%)
G0	1	41,914,250	20,957,125	35,506,898 (84.71%)	43,351,070	36,583,507	84.39
	2	44,423,646	22,211,823	37,674,222 (84.81%)			
	3	43,715,314	21,857,657	36,569,401 (83.65%)			
G1	1	42,443,636	21,221,818	35,442,650 (83.51%)	42,675,702	35,772,242	83.82
	2	44,392,916	22,196,458	37,298,311 (84.02%)			
	3	41,190,554	20,595,277	34,575,765 (83.94%)			
G2	1	40,378,914	20,189,457	34,083,517 (84.41%)	40,665,685.33	34,093,629	83.84
	2	40,932,238	20,466,119	34,090,846 (83.29%)			
	3	40,685,904	20,342,952	34,106,524 (83.83%)			
G3	1	53,099,790	26,549,895	44,666,920 (84.12%)	51,522,379.33	43,153,440.33	83.76
	2	51,901,662	25,950,831	43,334,048 (83.49%)			
	3	49,565,686	24,782,843	41,459,353 (83.65%)			
G4	1	46,540,170	23,270,085	38,908,154 (83.60%)	49,058,894	41,193,441	83.97
	2	47,105,492	23,552,746	39,399,685 (83.64%)			
	3	53,531,020	26,765,510	45,272,484 (84.57%)			

### RNA-seq data analysis

The files of raw reads were cleaned by removing adapter sequences and the clean reads were aligned onto the apple reference genome (https://iris.angers.inra.fr/gddh13//) [24]. An average of 83.95% of reads were mapped for each sample (Table 1). All usable reads were then calculated and normalized by an absolute value of log_2_ (fold change) with Fragments Per Kilobase of transcript per million mapped reads (FPKM) ≥ 1.0 was used as a threshold to determine significant differentially expressed genes (DEGs) [28]. A Gene Ontology (GO) database (http://www.geneontology.org) was used to assign apple genes to GO categories [29]. Besides, a Kyoto Encyclopedia of Genes and Genomes (KEGG) database (https://www.genome.jp/kegg/pathway.html) was used for KEGG pathway analyses. GO terms coupled with *KS*<0.01 was considered significantly enriched by DEGs (Additional file 1: Table S1). KEGG pathways with a *Q*-value ≤ 0.05 were considered significantly enriched (Table 2).

**Table 2:** Significantly enriched pathways of DEGs in ‘Red Delicious’ and its four generation mutants

Pathway ID	Pathway	Number of DEGs	Enrichment factor	*Q*-value (<0.05)
	G0 versus G1			
ko04626	Plant-pathogen interaction	14	3.97	0.000516177001036455
ko04075	Plant hormone signal transduction	16	3.46	0.000621209033772607
ko00909	Sesquiterpenoid and triterpenoid biosynthesis	4	9.64	0.0409888977775863
	G0 versus G2			
ko00941	Flavonoid biosynthesis	16	8.82	1.55373225396715e-09
ko04626	Plant-pathogen interaction	25	3.08	3.9124668653967e-05
ko04712	Circadian rhythm - plant	9	4.96	0.00618212628781434
ko04075	Plant hormone signal transduction	24	2.26	0.0120396678678723
ko00909	Sesquiterpenoid and triterpenoid biosynthesis	6	6.28	0.027494105974142
	G0 versus G3			
ko04075	Plant hormone signal transduction	53	2.60	7.99624033653856e-09
ko00941	Flavonoid biosynthesis	15	4.31	0.000139485985032328
ko00909	Sesquiterpenoid and triterpenoid biosynthesis	10	5.46	0.000908007889189655
ko04626	Plant-pathogen interaction	34	2.18	0.00135034354389485
ko04712	Circadian rhythm - plant	12	3.45	0.0158793441067531
ko00900	Terpenoid backbone biosynthesis	12	3.16	0.0371242613055638
	G0 versus G4			
ko00196	Photosynthesis - antenna proteins	11	6.29	6.37521150037568e-05
ko04712	Circadian rhythm - plant	15	3.84	0.000640829801345832
ko00941	Flavonoid biosynthesis	15	3.84	0.000640829801345832
ko00900	Terpenoid backbone biosynthesis	15	3.51	0.00196937336544101
ko04075	Plant hormone signal transduction	44	1.92	0.00212458752282485
	G1 versus G2			
ko00944	Flavone and flavonol biosynthesis	2	173.44	0.00100183440108559
ko00941	Flavonoid biosynthesis	4	18.26	0.00125854608191001
	G2 versus G3			
ko04075	Plant hormone signal transduction	22	3.04	0.000165774159370358
	G3 versus G4			
ko04075	Plant hormone signal transduction	16	4.21	4.46200952750608e-05

### Common expression pattern clustering analysis of DEGs

The different expression patterns of DEGs among the five strains were analysed using the R language, Cluster package, Biobase package, and *Q*-value package. The DEGs with a common expression trend were divided into a data set, which was expressed as a model map. The distance measure used was Euclidean distance, and the clustering method was K-means clustering or hierarchical clustering.

### Correlation analysis

A correlation matrix was prepared using SPSS statistical software and Pearson’s correlation coefficient as the statistical metric. The analysis was performed using the anthocyanin content at S2 and the FPKM average of each candidate DEG. Correlation values were converted to distance coefficients to define the height scale of the dendrogram.

### Quantitative reverse-transcription PCR (qRT-PCR) validation of RNA-Seq data

DNase-treated RNA (2 μg) was reverse transcribed in a reaction volume of 20 μl using PrimerScript^TM^ RT reagent Kit with gDNA Eraser (Takara, Dalian, China). Gene-specific primers were designed using Primer Express software (Applied Biosystems) (Additional file 2: Table S2). qRT-PCR assays were performed with SYBR Green PCR Master Mix (Takara, Dalian, China). The qRT-PCR reaction solution (20 μL total volume) was composed of 1.0 μL of each primer, 2 μL of cDNA, 10.0 μL 2×SYBR Green Master Mix, and 6.0 μL RNase-free water. Three biological and three technical replicates for each reaction were analysed on a LightCycler® 96 SW 1.1 instrument (Roche). The amplification program was described in detail by Mao et al. (2017) [27]. All relative expression was normalized by comparing with G0 expression and analysed using the comparative 2^-ΔΔC^T method [30] against the reference gene *MdGADPH*.

### Statistical analysis

Data regarding the anthocyanin content and the relative expression level of specific genes were analysed by ANOVA. SPSS 21.0 software was used for statistical analysis of treatment means, which were separated by Duncan’s multiple range test at *P* < 0.05. For correlation analysis, the Pearson correlation coefficient (*r*) was calculated, and a two-tailed test was carried out.

## Results

### Fruit skin pigmentation increased with bud sport generation and maturation

Visual inspection of apple skin colour during development revealed that the G2, G3 and G4 strains began colouring at S1, with the most visibly intense red colouring occurring in G3 and G4 (Fig. 1a). Pigmentation of these five strains progressively advanced to much higher levels from S1 to the subsequent stage S3, resulting in red fruit at maturity. In addition, red coloration gradually proliferated from G0 to G4 during the three stages.

Consistent with visual inspection, the analysis of apple skin anthocyanin contents showed that the levels of total anthocyanins in the five strains were initially low and sharply increased with maturation (from S1 to S3) (Fig. 1b). Relative to that at G0, the total anthocyanin level in fruit skin was ~0.78-, ~1.20-, ~5.02- and ~2.85-fold higher in G1, G2, G3 and G4 at stage S1, respectively. At S2, the level in fruit skin was ~1.41-, ~4.65-, ~6.33- and ~4.72-fold higher in G1, G2, G3 and G4, respectively. Finally, at S3, the level in fruit skin was ~2.14-, ~3.41-, ~5.25- and ~4.35-fold higher in G1, G2, G3 and G4, respectively. Briefly, anthocyanin content was lowest in G0 and highest in G3, followed by the contents in G4, G2, and G1. It was concluded that the more intense red colouring pattern in the apple skin of bud sport mutants was mainly caused by the accumulation of anthocyanins. In addition, a more blushed colouring pattern was observed with an increase in the number of bud sport generations. However, the third-generation mutant G3 showed greater blushing than did the fourth-generation mutant G4 at S1 and S2, and there was no significant difference at S3.

### The contents of IAA and ABA in apple skin increased with bud sport generation at color break stage, while the content of GA decreased

Hormone levels were also analyzed at three time points (S1, S2, and S3). The overall trend of IAA concentrations in apple skin first increased and then decreased from S1 to S3 and peaked at S2 (Fig. 1c). However, the GA content decreased from S1 to S3 (Fig. 1d). ABA concentrations of G0, G1 and G2 peaked at S1, whereas those of G3 and G4 peaked at S2 (Fig. 1e). Importantly, the IAA and ABA contents of G3 and G4 in apple skin at color break stage S2 were considerably higher than those of G2, while G0 and G1 showed considerably lower levels than did G2. Nevertheless, GA concentrations, which displayed a trend opposite that of IAA and ABA, decreased with bud sport generation, that is, from G0 to G4.

### Transcriptomic profiling of the fruit skin from ‘Red Delicious’ and its four continuous generation mutants

Triplicate sampling of the fruit skin from ‘Red Delicious’ and its four continuous generation mutants at S2 yielded 15 RNA samples for RNA sequencing (RNA-seq) analysis, and the mapping rate of 20,189,457-26,765,510 clean reads onto the apple reference genome (https://iris.angers.inra.fr/gddh13//) (Daccord et al. 2017) ranged from 83.29% to 84.81% (Table 1). The average number of mapped reads ranged from 34,093,629 in G2 to 43,153,440.33 in G3. An FDR < 0.01 and fold changes ≥ 2 were used as screening criteria for DEGs; in addition, an FPKM (fragments per kilobase of mapped reads) ≥ 1.0 in at least one of the 5 triplicate samples was considered to be expressed. The mean normalized expression value (FPKM) per transcript of the three biological replicates was calculated for each sample using the geometric normalization method. The resulting dataset comprising 33,192 transcripts was used for subsequent analysis (Additional file 3: Dataset S1).

### Differentially expressed genes (DEGs) in ‘Red Delicious’ versus its mutants gradually increased with bud sport generation at color break stage

**Fig. 2:**
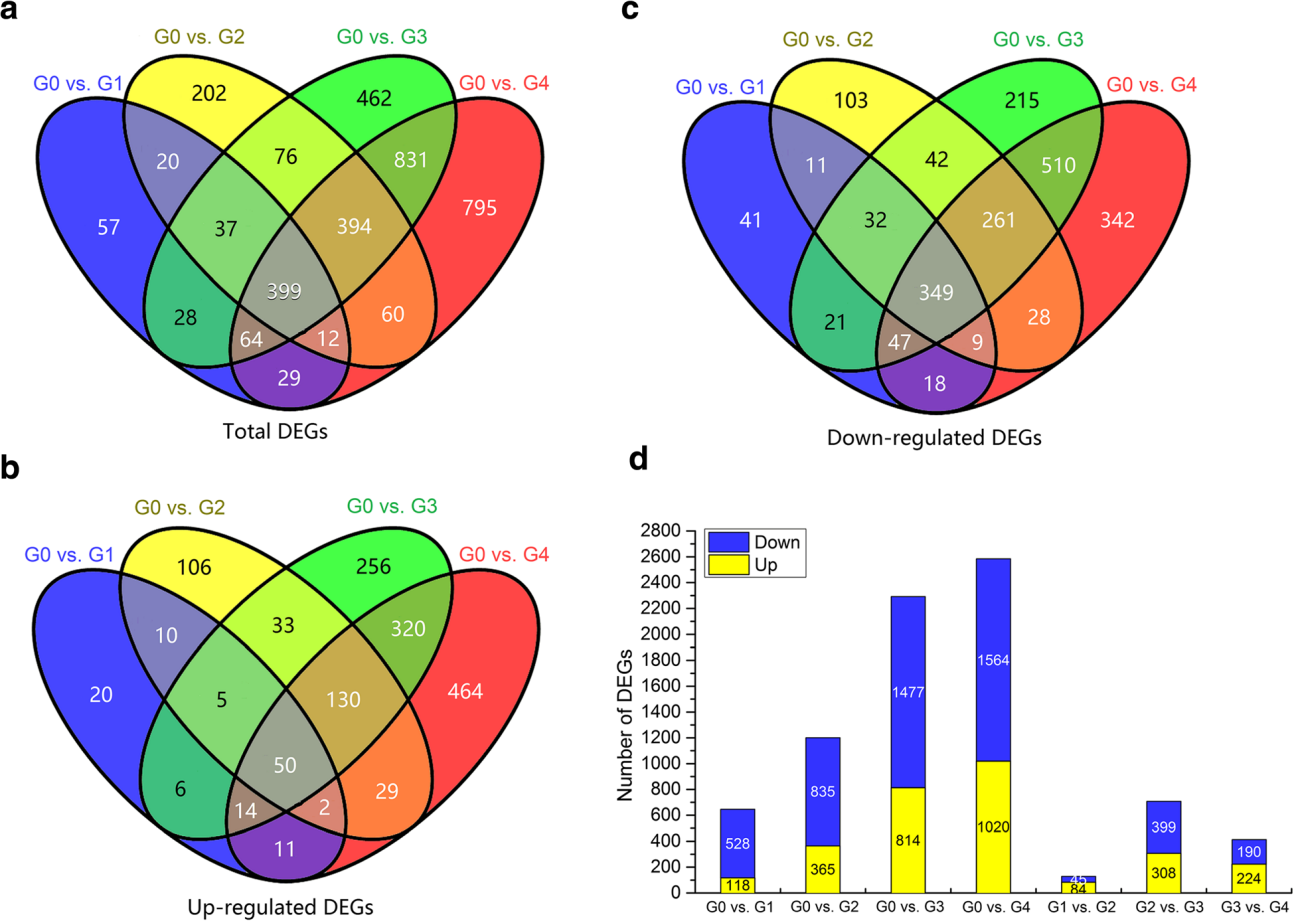
Summary of the number of differentially expressed genes (DEGs) identified by RNA-seq analysis in the fruit skin tissues of ‘Red Delicious’ and its four generation mutants (‘Starking Red’, ‘Starkrimson’, ‘Campbell Redchief’ and ‘Vallee spur’) at S2, named G0 to G4. The number of total DEGs (**a**), up-regulated DEGs (**b**), and down-regulated DEGs (**c**) are presented by Venn diagrams (FDR < 0.01 and fold change ≥ 2). **d**, Number of total up-regulated and down-regulated DEGs. The histogram represents the number of commonly down-regulated (blue) and up-regulated (yellow) DEGs

To identify DEGs in ‘Red Delicious’ and its four generation mutants, seven pairwise transcriptome comparisons (i.e., G0 vs. G1, G0 vs. G2, G0 vs. G3, G0 vs. G4, G1 vs. G2, G2 vs. G3, and G3 vs. G4) were performed at S2 (Fig. 2, Additional file 4: Figure S1 and Additional file 5: Table S3). The total number of DEGs was 3,466, including 1,456 up-regulated DEGs and 2029 down-regulated DEGs (Fig. 2 and Additional file 6: Dataset S2). Among them, the number of both up-regulated and down-regulated DEGs increased considerably from the first generation mutant G1 to the fourth generation mutant G4 versus the number observed in G0, and the smallest number was observed in G1 versus that in G2. Moreover, we found that more genes were down-regulated than up-regulated in G1, G2, G3 and G4 relative to G0.

### Comparative transcriptome enrichment analysis identified key processes responsible for anthocyanin accumulation in ‘Red Delicious’ and its mutants

To understand the major functional categories represented by the DEGs, GO functional enrichment analysis was carried out using all reference genes as background. The results of GO functional enrichment analysis are displayed in three main categories: Biological process, cellular component and molecular function (Additional file 1: Table S1). The biological process category was significantly enriched into three terms, namely, defence response to fungus, 1-aminocyclopropane-1-carboxylate biosynthetic process and negative regulation of growth, were observed in G0 vs. G1, G0 vs. G2, G0 vs. G3 and G0 vs. G4; among these terms, 1-aminocyclopropane-1-carboxylate biosynthetic process and negative regulation of growth were also enriched in G1 vs. G2, G2 vs. G3 and G3 vs. G4. Furthermore, in addition these two GO terms, three other significantly enriched GO terms, namely, chitin catabolic process, regulation of leaf development and DNA conformation change, were shared in G0 vs. G1, G1 vs. G2, G2 vs. G3 and G3 vs. G4. In the cellular component category, GO term of elongator holoenzyme complex was enriched in G0 vs. G1, G0 vs. G2, G0 vs. G3 and G0 vs. G4, whereas, elongator holoenzyme complex, mitochondrial intermembrane space and U12-type spliceosomal complex were shared in G0 vs. G1, G1 vs. G2, G2 vs. G3 and G3 vs. G4. In the molecular function category, nine terms were observed in the seven abovementioned comparison groups: ADP binding, L-iditol 2-dehydrogenase activity, acyl-CoA hydrolase activity, 3-beta-hydroxy-delta5-steroid dehydrogenase activity, O-methyltransferase activity, 1-aminocyclopropane-1-carboxylate synthase activity, naringenin-chalcone synthase activity, catechol oxidase activity and trans-cinnamate 4-monooxygenase activity. Moreover, chitinase activity, sulfur compound binding and caffeate O-methyltransferase activity were only shared in G0 vs. G1, G1 vs. G2, G2 vs. G3 and G3 vs. G4.

To further systematically understand the molecular interactions among the DEGs, we performed KEGG analysis using KOBAS software. The significantly enriched KEGG pathway term sesquiterpenoid and triterpenoid biosynthesis was shared in G0 vs. G1, G0 vs. G2 and G0 vs. G3, but not in G0 vs. G4 (Table 2). However, the sesquiterpenoid and triterpenoid biosynthesis pathway was derived from terpenoid backbone biosynthesis, which occurred both in G0 vs. G3 and G0 vs. G4. Furthermore, the number of DEGs belonging to the triterpenoid biosynthesis pathway gradually increased from G0 vs. G1 to G0 vs. G4. In addition, the flavonoid biosynthesis pathway was enriched in G0 vs. G2, G0 vs. G3 and G0 vs. G4. Moreover, the plant hormone signal transduction pathway was enriched in G0 vs. G1, G0 vs. G2, G0 vs. G3, G0 vs. G4, G2 vs. G3 and G3 vs. G4 but not in G1 vs. G2. Thus, the four abovementioned candidate pathways were considered to be heavily involved in anthocyanin accumulation.

### Functional classification of DEGs in ‘Red Delicious’ and its four continuous generation mutants

**Fig. 3:**
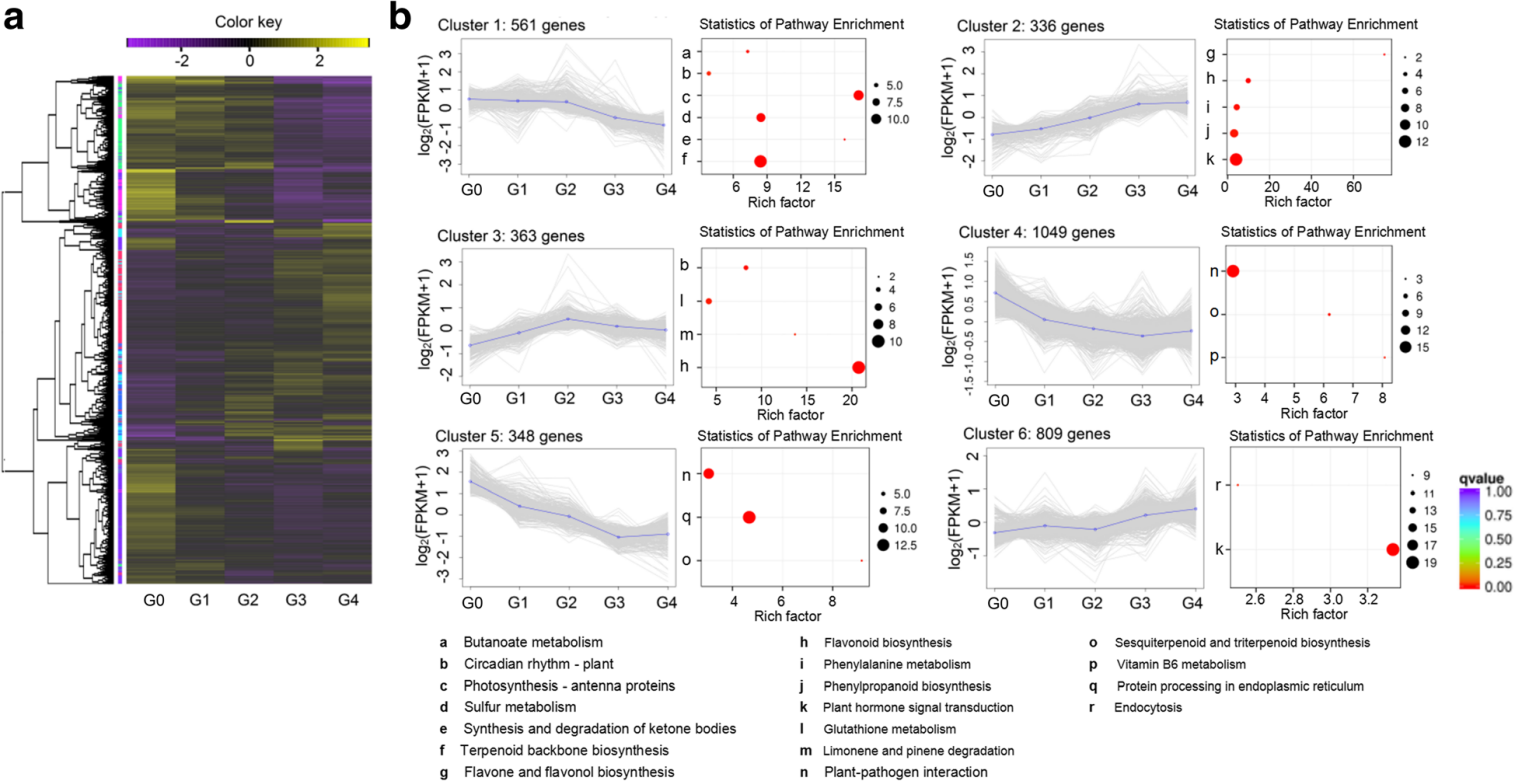
Gene expression profiles and KEGG category distribution of the DEGs in the six common expression clusters composing the fruit skin transcriptome at S2. Clusters were derived by coupled clustering analysis of the 3,466 commonly modulated genes. **a** Heat map of the overall common expression pattern. **b** Each line represents the log_2_-transformed average of the mean FPKM values for an individual transcript. Significantly overrepresented KEGG categories are represented by red dots. KEGG category enrichment was computed using the R language, Cluster package, Biobase package, and *Q*-value package (*P* ≤ 0.01)

To further identify the major functions of DEGs and establish the skin pigment transcriptome, clustering analysis was applied to the 3,466 DEGs. These genes were grouped into six expression patterns (Fig. 3 and Additional file 7: Dataset S3). Cluster 1 contained 561 DEGs whose expression peaked at G0/G1/G2. Cluster 2 contained 336 DEGs whose expression peaked at G3/G4. Cluster 3 contained 363 DEGs whose expression peaked at G2. Furthermore, 1049 and 348 DEGs whose expression peaked at G0 were included in clusters 4 and 5, respectively. Cluster 6 contained 809 DEGs whose expression peaked at G4. KEGG analysis was also carried out for DEGs belonging to each pattern with a *P*-value ≤ 0.01. The expression pattern of cluster 2 was positively consistent with total anthocyanin content (Fig. 1b), whereas clusters 4 and 5 were negatively aligned. DEGs in cluster 2 were significantly enriched in plant hormone signal transduction, flavonoid biosynthesis, flavone and flavonol biosynthesis, phenylalanine metabolism, and phenylpropanoid biosynthesis. Among those, some of the final metabolites of flavonoid biosynthesis, flavone and flavonol biosynthesis, phenylalanine metabolism, and phenylpropanoid biosynthesis are anthocyanins (Massonnet et al. 2017). Remarkably, the pathway that was co-enriched by clusters 4 and 5 was sesquiterpenoid and triterpenoid biosynthesis, which was hypothesized to be negatively related to the accumulation of anthocyanins. In addition, the pathway of terpenoid backbone biosynthesis was also enriched in cluster 1, and flavonoid biosynthesis and phenylalanine metabolism were enriched in cluster 3. Overall, the results of the functional classification analysis of the common expression patterns of DEGs combined with KEGG agreed with the results of the aforementioned comparative transcriptome enrichment analysis. Therefore, pathways including flavonoid/phenylpropanoid biosynthesis, terpenoid biosynthesis and plant hormone signal transduction were selected for subsequent analysis.

### Key candidate DEGs responsible for anthocyanin accumulation in ‘Red Delicious’ and its mutants

#### Genes involved in phenylpropanoid/flavonoid biosynthesis pathway

**Fig. 4:**
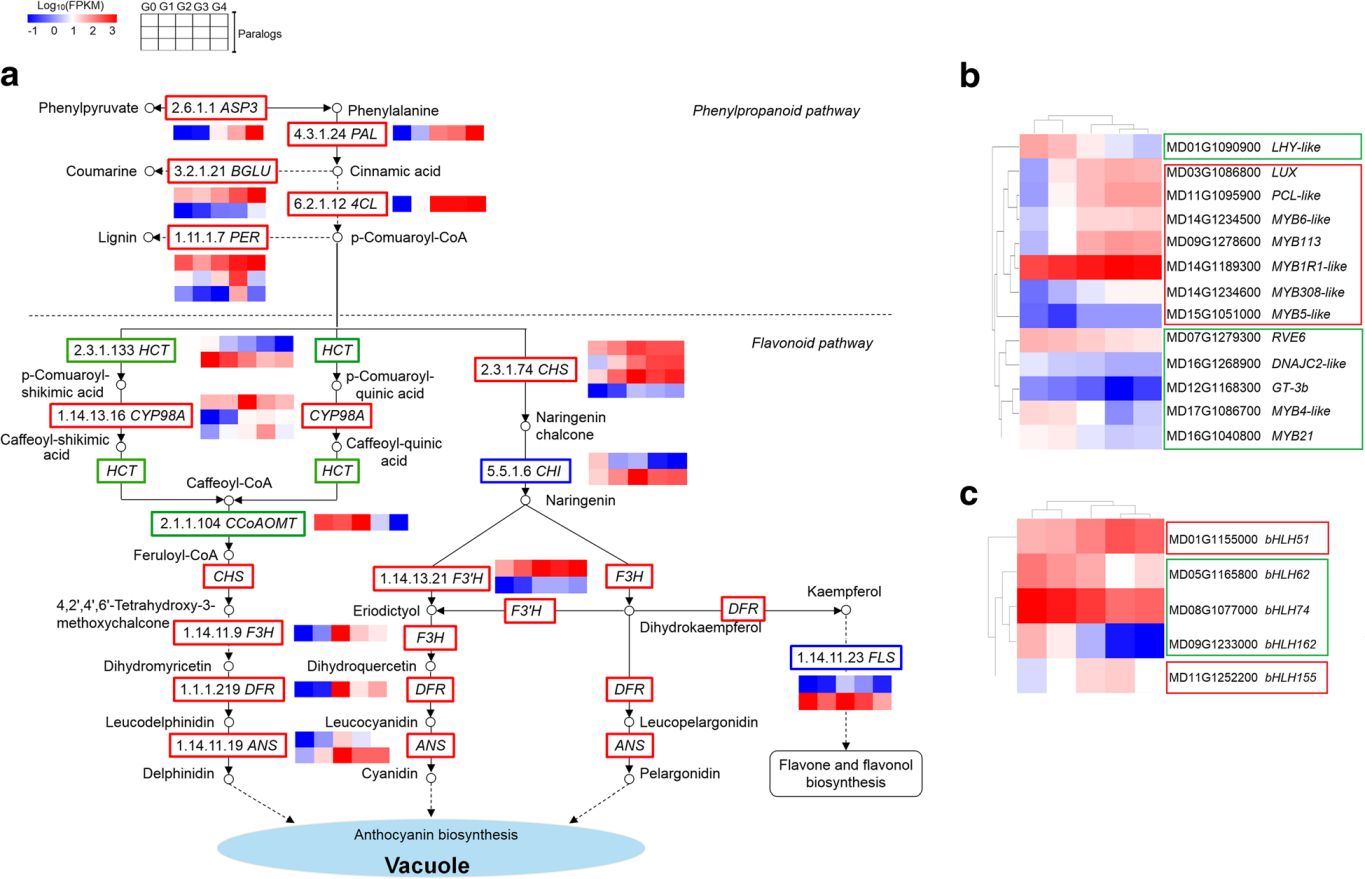
**a** Differential expression of genes involved in phenylpropanoid/flavonoid biosynthesis pathway hormone signal transduction pathway in ‘Red Delicious’ and its four generation mutants (‘Starking Red’, ‘Starkrimson’, ‘Campbell Redchief’ and ‘Vallee spur’), named G0 to G4. Differential expression of transcription factors that encode MYB-like (**b**) and helix-loop-helix (**c**) DNA-binding domains. Heat maps depict the normalized gene expression values, which represent the means ± SD of three biological replicates. Expression values of five libraries are presented as FPKM normalized log_10_-transformed counts

Variety-specific trends in the expression of phenylpropanoid/flavonoid biosynthesis pathway genes at S2 were investigated by preparing heat maps (Fig. 4a). We focused on the 28 DEGs involved in this pathway, including 4 from cluster 1, 13 from cluster 2, 10 from cluster 3, and 1 from cluster 5 (Additional file 8: Table S4). Differences in the expression pattern of these genes from cluster 2 were found among ‘Red Delicious’ and its four continuous generation mutants, closely mirroring the differences in total anthocyanin concentration at S2. For example, all DEGs involved in the phenylpropanoid biosynthesis pathway, including one aspartate aminotransferase cytoplasmic (*ASP3*), one phenylalanine ammonia-lyase (*PAL*), two beta-glucosidase (*BGLU*), one 4-coumarate-CoA ligase (*4CL*) and three peroxidases (*PER*), were from cluster 2, demonstrating a gradually increasing expression pattern from G0 to G4 (Fig. 4a and Additional file 9: Dataset S4). Among the DEGs, *ASP3* participates in the synthesis of phenylalanine and phenylpyruvate, which are precursors of anthocyanin synthesis. *BGLU* and *PER* are involved in coumarine and lignin biosynthesis, respectively. *PAL* and *4CL* are phenylpropanoid genes. In addition, down-regulated genes, including two quinate hydroxycinnamoyl transferase (*HCT*) and one caffeoyl-CoA O-methyltransferase (*CCoAOMT*), and up-regulated genes, including three cytochrome P450 98A2-like (*CYP98A*), four *CHS*, one avanone-3-hydroxylase (*F3H*), one dihydroflavonol reductase (*DFR*), and one anthocyanidin synthase (*ANS*), are involved in the synthesis of delphinidin from the phenylpropanoid biosynthesis pathway. Furthermore, the four *CHS* genes described above, one down-regulated and one up-regulated *CHI* gene, two *F3’H* genes, and the aforementioned *F3H*, *DFR* and *ANS* are involved in the synthesis of cyanidin. *F3’H* and other genes involved in pelargonidin synthesis are the same as those involved in cyanidin synthesis. These findings confirm that delphinidin, cyanidin and pelargonidin are the three main substances responsible for the synthesis of anthocyanins via the phenylpropanoid biosynthesis pathway in apple. Moreover, two down-regulated *FLS* genes are involved in flavone and flavonol biosynthesis.

#### Genes involved in MYB-like and helix-loop-helix DNA-binding domain transcriptional factors

Correlative analysis was carried out between anthocyanin content at S2 and the expression levels of transcription factors that encode MYB-like and helix-loop-helix DNA-binding domains in DEGs (Additional file 10: Dataset S5 and Additional file 11: Dataset S6). As a result, 13 MYB and 5 bHLH transcription factors were screened. Among them, the expression of MYB members, including *LUX*, *MYB113*, *PCL*-*like*, *MYB1R1*-*like*, *MYB6*-*like*, *MYB308*-*like* and *MYB5*-*like*, bHLH members, including *bHLH51* and *bHLH155*, presented a notable positive correlation with anthocyanin content at S2 (*P*<0.05) (Fig. 4b, c). MYB members *LHY-like*, *RVE6*, *GT-3b*, *MYB21*, DNAJC2-like and *MYB4-like* and bHLH members *bHLH62*, *bHLH74* and *bHLH162* showed a remarkably negative correlation with anthocyanin content at color break stage S2 (*P*<0.05).

#### Genes involved in terpenoid biosynthesis and plant hormone signal transduction

**Fig. 5:**
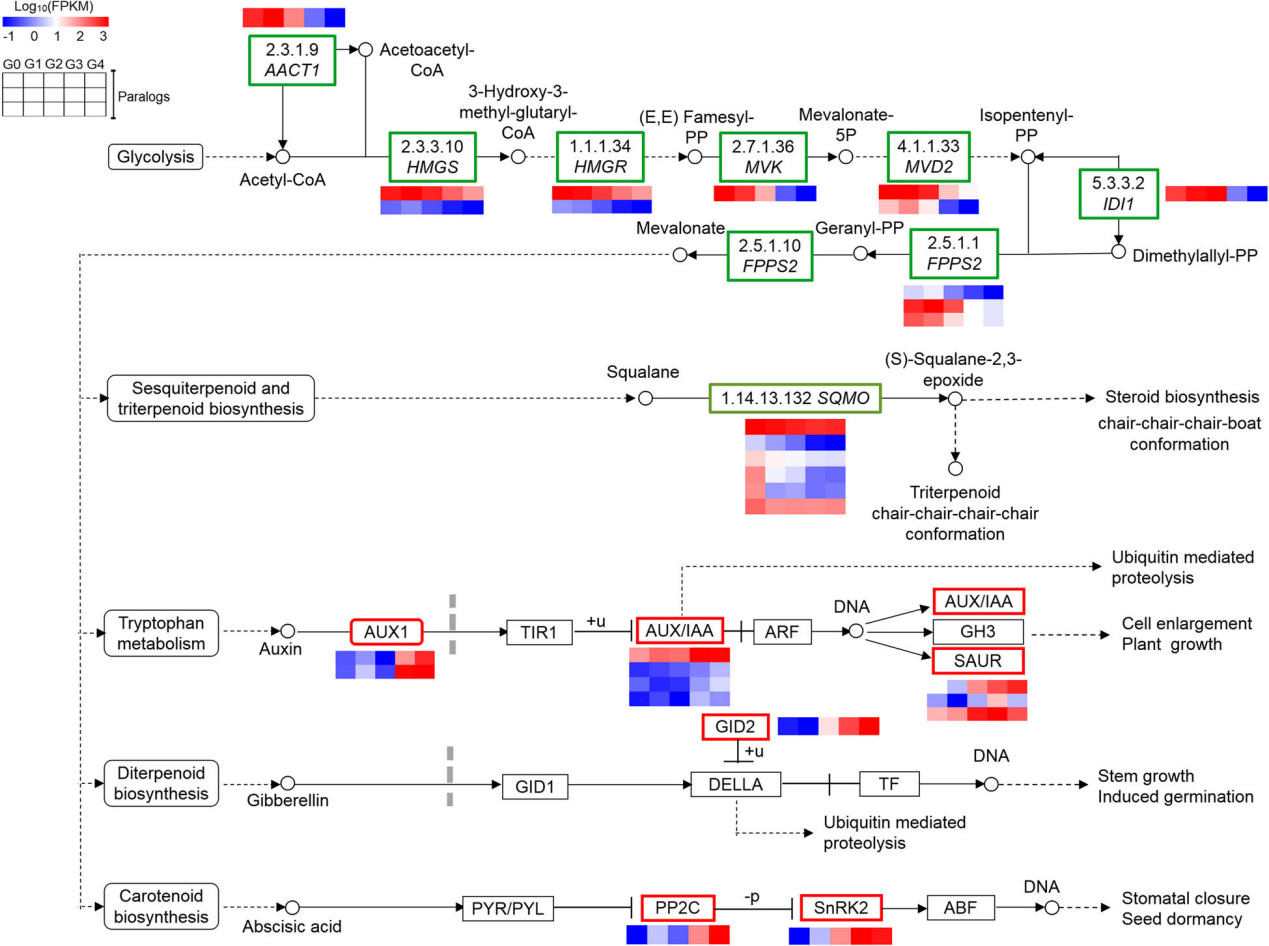
Differential expression of genes involved in sesquiterpenoid and triterpenoid/terpenoid biosynthesis coupled with hormone signal transduction pathway in ‘Red Delicious’ and its four generation mutants (‘Starking Red’, ‘Starkrimson’, ‘Campbell Redchief’ and ‘Vallee spur’), named G0 to G4. Heat maps depict the normalized gene expression values, which represent the means ± SD of three biological replicates. Expression values of five libraries are presented as FPKM normalized log_10_-transformed counts

KEGG analysis showed that terpenoid backbone biosynthesis was over-presented in cluster 1 and that sesquiterpenoid and triterpenoid biosynthesis branching from terpenoid backbone biosynthesis was over-presented in clusters 4 and 5 (Fig. 3), indicating that this pathway may play important roles in anthocyanin accumulation in apple. Moreover, the plant hormone signal transduction pathway, which is modulated by terpenoid biosynthesis, was enriched in cluster 2. A total of 18 DEGs in the terpenoid biosynthesis pathway (Additional file 12: Dataset S7 and Additional file 13: Table S5) and 12 DEGs in the plant hormone signal transduction pathway (Additional file 14: Dataset S8 and Additional file 15: Table S6) were also investigated by preparing heat maps (Fig. 5). The expression of DEGs involved in terpenoid backbone biosynthesis, including one acetyl-CoA acetyltransferase, cytosolic 1 (*AACT1*), two hydroxymethylglutaryl-CoA synthase-like (*HMGS*), two 3-hydroxy-3-methylglutaryl-coenzyme A reductase 1-like (*HMGR*), one mevalonate kinase-like (*MVK*), two diphosphomevalonate decarboxylase MVD2-like (*MVD2*), one isopentenyl-diphosphate Delta-isomerase I (*IDI1*), three farnesyl pyrophosphate synthase 2-like (*FPPS2*) and six squalene monooxygenase-like (*SQMO*), were gradually down-regulated from G0 to G4. Nevertheless, two auxin transporter-like 1 (*AUX1*), four auxin-responsive protein *AUX/IAA* and three auxin-responsive protein *SAUR* involved in tryptophan metabolism of auxin biosynthesis; one F-box protein GID2-like (*GID2*) involved in diterpenoid biosynthesis of GA biosynthesis; and one protein phosphatase 2C (*PP2C*) and one sucrose non-fermenting-1-related protein kinase 2 (*SnRK2*) involved in carotenoid biosynthesis of ABA biosynthesis were up-regulated from G0 to G4.

**Fig. 6:**
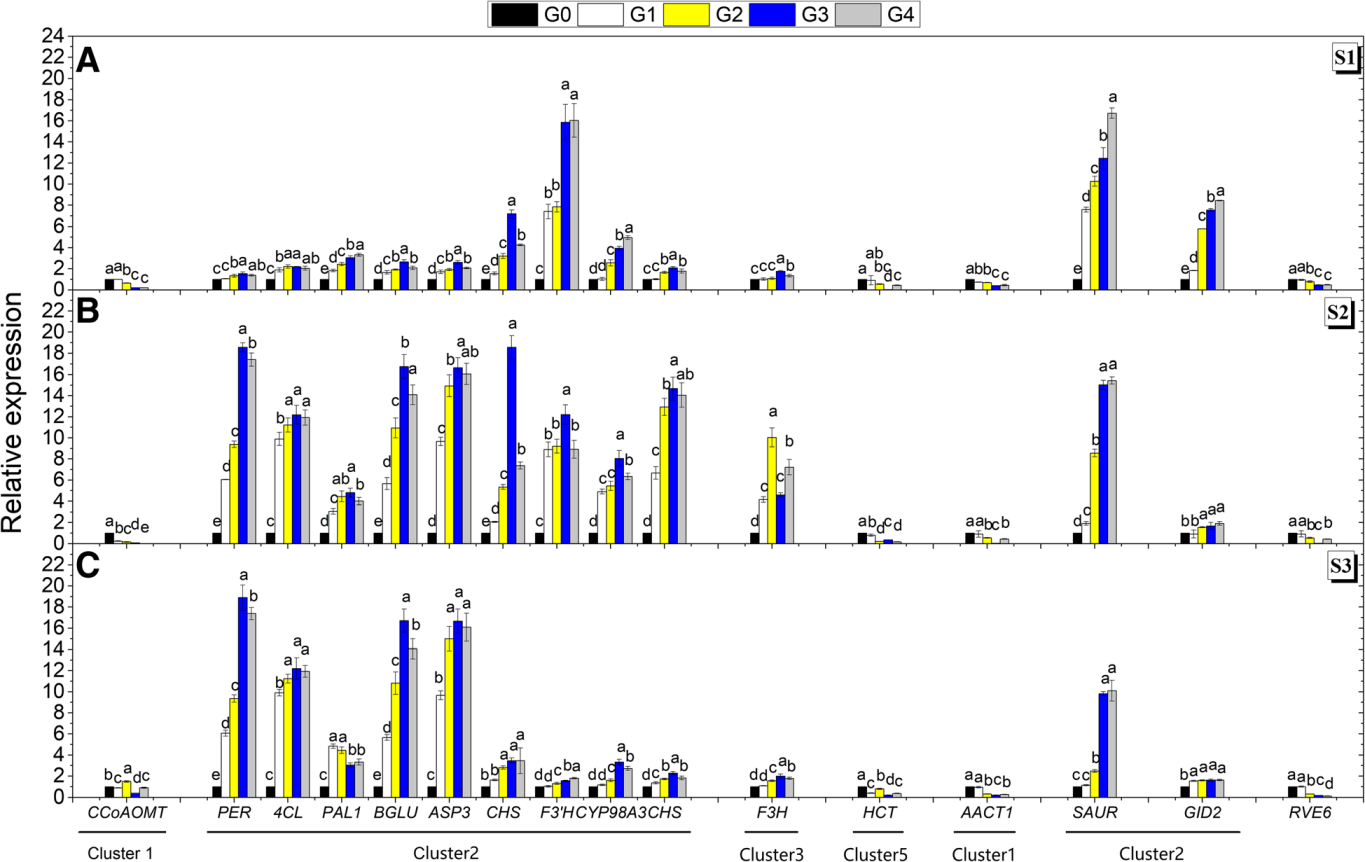
Relationships between total anthocyanin contents and transcript levels of the sixteen representative genes from Figs. 4 and 5. For each accession, the expression was determined in three developmental stages (S1 to S3) of fruit skin tissues. For the qRT-PCR assay, the mean was calculated from three biological replicates, each with three technical replicates (*n*=9). These replicates were then normalized relative to the expression of *MdGADPH*. The x-axis in each chart is the same and represents different Malus accessions, as indicated by names in the bottom panel, which are arranged in different clusters. The left y-axis represents relative expression levels determined by qRT-PCR

To further evaluate the validity of our results, 16 representative DEGs used previously as shown in Figs. 4 and 5 were selected for expression level examination by qRT-PCR (Additional file 2: Table S2). The overall trend of relative expression levels at three stages was consistent with that of deep sequencing at S2 (Fig. 6), suggesting that the candidate genes involved in pathways such as phenylpropanoid/flavonoid biosynthesis, terpenoid biosynthesis and plant hormone signal transduction, appended with MYB and bHLH transcriptional factors, were directly correlated with anthocyanin accumulation.

## Discussion

### The activation of early phenylpropanoid biosynthesis genes was more responsible for anthocyanin accumulation in apple skin of bud sport mutants

In plants, phenylpropanoid biosynthesis gives rise to a large number of secondary metabolites, including hydroxycinnamic acids, monolignols/lignin, coumarins, benzoic acids, stilbenes, anthocyanins and flavonoids, serving different functions in plant development, reproduction, defence, and protection against biotic/abiotic stresses [31–33]. Differences in the expression pattern of genes involved in phenylpropanoid/flavonoid biosynthesis result in diverse anthocyanin profiles [2, 25]. Our survey provided a comprehensive profile of the phenylpropanoid/flavonoid biosynthesis pathway in ‘Red Delicious’ and its four continuous generation mutants. The results showed that all of the early phenylpropanoid biosynthesis pathway genes, including *ASP3*, *PAL*, *4CL*, *BGLU* and *PER*, were aggregated in cluster 2 (Fig. 4a and Additional file 8: Table S4), which matched the anthocyanin content (Fig. 1a, b). Other genes in cluster 2 containing *CHS*, *CYP98A* and *F3’H* are involved in the middle steps of the phenylpropanoid pathway, that is, the early steps of the flavonoid biosynthesis pathway. Nevertheless, genes encoding *CHS*, *CYP98A*, *CHI*, *F3H*, *DFR*, *FLS* and *ANS* were involved in the middle and late steps of the phenylpropanoid biosynthesis pathway and gathered in clusters 3, which was contrary to the findings in previous reports where the late biosynthetic genes usually correlated positively with the anthocyanin content [34–37]. Remarkably, anthocyanin concentrations of glucose-treated *Paeonia suffruticosa* ‘Luoyang Hong’ cut flowers were higher than those of the sucrose-treated flowers and increased significantly from the pre-opening stage to the full opening stage, whereas the expression of *PsCHI1*, *PsF3H1* and *PsDFR1* was differentially induced by sucrose and glucose at different development stages [38]. This report is in line with our results which shows that the expression levels of *F3H* which was selected for qRT-PCR verification from cluster 3 were consistent with the total anthocyanin contents at the corresponding stages, S1 and S3 (Fig. 6). In addition, members of *CHS* were also enriched in cluster 1, 2 and 3. Similarly, transcript levels of *BrCHS1*, -*4*, -*5*, *F3H*, *DFR*, and *ANS* were high in pigmented epidermis of light-exposed swollen hypocotyls from *Brassica rapa*, while those of *BrCHS2*, -*3*, and -*6* were almost undetectable [39]. *MdUFGT2* which was involved in the late step of the phenylpropanoid biosynthesis pathway was up-regulated only in non-red apple cultivars, while *MdUFGT4* was up-regulated only in red skin cultivar [40]. In this case, different gene family members encoding structural genes were expressed at different levels and these need to be further investigate in future. To sum up, the activation of early phenylpropanoid biosynthesis pathway genes was demonstrated to be most responsible for pigment accumulation in the apple skin of bud sport mutants at the color break stage. In addition, *ASP3*, *BGLU* and *PER* were confirmed to be involved in the synthesis of phenylpyruvate, coumarine and lignin, respectively (Fig. 4a). Interestingly, 44 stilbene synthase (*STS*) genes involved in stilbene biosynthesis were characterized to influence anthocyanin accumulation during grapevine (*Vitis vinifera*) maturation as reported by Massonnet et al. (2017) [25]. Nevertheless, these genes do not exist among our 3,466 DEGs, possibly because they are variety-specific in nature [41].

### MYB and bHLH modulated anthocyanin accumulation in apple skin by regulating the transcription of genes involved in the phenylpropanoid/flavonoid pathway

MYB and bHLH autonomously mediated the transcription of genes involved in the middle steps of the phenylpropanoid pathway, that is, the early steps of the flavonoid biosynthetic pathway (*CHS*, *CHI*, *F3H*, *F3’H* and *FLS*), which leads to the production of colourless dihydroflavonol compounds [20, 42–45]. The heterologous expression of *OjMYB1* in Arabidopsis could enhance anthocyanin content and up-regulate the expression levels of structural gene-related anthocyanin biosynthesis [46]. The red radish (*Raphanus sativus* L.) bHLH transcription factor RsTT8 acts as a positive regulator of anthocyanin biosynthesis [47]. Nevertheless, *AtMYB4*, *AmMYB308*, *FaMYB1*, *ZmMYB31*, *ZmMYB42*, *PhMYB4, VvMYBC2-L1* and *PtrMYB57* have been demonstrated to repress phenylpropanoid synthesis, likely via repression of synthesis genes [48–52]. We corroborated that MYB members, including *LUX*, *MYB113*, *PCL-like*, *MYB1R1-like*, *MYB6-like*, *MYB308-like* and *MYB5-like*, and bHLH members, including *bHLH51* and *bHLH155*, showed a notable positive correlation with anthocyanin content (Additional file 10: Dataset S5, Additional file 11: Dataset S6 and Fig. 4b, c) and were considered to promote anthocyanin synthesis by mediating the transcription of structural genes, *CHS* and *F3’H*, which are involved in the flavonoid pathway. Other MYB members, including *LHY-like*, *RVE6*, *GT-3b*, *MYB21*, *DNAJC2-like* and *MYB4-like*, and bHLH members, including *bHLH62*, *bHLH74* and *bHLH162* showed a remarkably negative correlation with anthocyanin content and were demonstrated to repress anthocyanin synthesis. In addition, HD-Zip I transcription factor MdHB1 was involved in the regulation of anthocyanin accumulation [53]. When MdHB1 is silenced, *MdMYB10*, *MdbHLH3*, and *MdTTG1* are released to activate the expression of *MdDFR* and *MdUFGT* and anthocyanin biosynthesis, resulting in red flesh in apple cv. ‘Granny Smith’ [54]. The expression of *F3’5’H*, *DFR* and *ANS* is strongly inhibited by the increase in the expression of *MYBL1*, which is a novel R3 MYB transcription factor classified as an MYB transcriptional repressor [55]. However, a full understanding of the mechanism by which structural genes involved in anthocyanin synthesis are specifically mediated by MYB and bHLH remains elusive clear and requires further investigation.

### Terpenoid biosynthesis modulated anthocyanin accumulation by positively regulating plant hormone signal transduction in apple skin of bud sport mutants

Hormones are important factors inducing anthocyanin accumulation [56–58]. Carvalho et al. (2010) [12] provided evidence that anthocyanin accumulation was promoted by exogenous ABA and CTK and inhibited by GA in tomato hypocotyls. Co-treatment of IAA and CTK significantly enhanced the cytokinin-induced increase in anthocyanin levels, but an auxin concentration that was too high strongly inhibited anthocyanin synthesis even in the presence of cytokinin in callus cultures of red-fleshed apple (*M. sieversii* f.*niedzwetzkyana*), as shown by Ji et al. (2014) [16]. Our results showed that sesquiterpenoid and triterpenoid biosynthesis along with plant hormone transduction, including tryptophan metabolism for IAA, diterpenoid biosynthesis for GA and carotenoid biosynthesis for ABA, branches from the general terpenoid backbone synthesis pathway and shares the same precursors as glycolysis (Fig. 5). The sesquiterpenoid and triterpenoid biosynthesis pathways comprised six *SQMO* genes in clusters 4 and 5, which were negatively correlated with anthocyanin content. The genes *AACT1*, *HMGS*, *HMGR*, *MVK*, *MVD2*, *IDI1* and *FPPS2*, involved in the early steps of terpenoid backbone synthesis, were in cluster 1, which also reflected the differential accumulation of anthocyanin to some extent. Moreover, *AUX1*, *AUX/IAA* and *SAUR*, associated with tryptophan metabolism for IAA; *GID2*, associated with diterpenoid biosynthesis for GA; and *PP2C* and *SnRK2*, associated with carotenoid biosynthesis for ABA, were in cluster 2, which positively reflected the anthocyanin content. Likewise, the contents of IAA and ABA increased, while GA decreased with maturation and pigment accumulation from G0 to the fourth-generation mutant G4 (Fig. 1c, e). Therefore, the down-regulation of genes involved in terpenoid biosynthesis positively induced the expression of *AUX1*, *AUX/IAA*, *SAUR*, *GID2*, *PP2C* and *SnRK2*, resulting in increased synthesis of IAA and ABA and decreased synthesis of GA, thus modulating anthocyanin accumulation. Loreti et al. (2010) [13] suggested the existence of crosstalk between the sucrose and hormone signalling pathways in the regulation of the anthocyanin biosynthetic pathway. Similarly, exogenous ABA promoted fruit ripening by increasing anthocyanin content in sweet cherry (*Prunus avium* L.) cv. Sato Nishiki, and the expression of *PaPP2C3*, *PaPP2C5* and *PaPP2C6* was significantly induced by exogenous ABA [58]. In general, there may be some form of crosstalk between the activation of phenylpropanoid biosynthesis and plant hormone signal transduction in pigment accumulation of apple bud sport mutants.

## Conclusions

We have investigated the fruit skin transcriptome of ‘Red Delicious’ and its four continuous generation mutants (‘Starking Red’, ‘Starkrimson’, ‘Campbell Redchief’ and ‘Vallee spur’) and identified specific processes that lead to the accumulation of anthocyanin. The results indicate that apple skin pigmentation and anthocyanin content were increased in the mutants due to bud sport. Terpenoid biosynthesis influences anthocyanin accumulation by positively regulating the synthesis of IAA and ABA and negatively regulating the synthesis of GA. MYB and bHLH modulate anthocyanin accumulation in apple skin by regulating the transcription of genes *CHS* and *F3’H*. *ASP3*, *CYP98A* and *CCoAOMT* are novel anthocyanin-associated genes of apple first reported the present study. This novel set of genes provides not only new insights into anthocyanin biosynthesis but also important clues for more dedicated studies to broaden our knowledge of the anthocyanin pathway in apple.

## Additional files

Additional file 1: **Table S1.** Gene ontology (GO) enrichment analyses for DEGs in ‘Red Delicious’ and its four generation mutants (XLS 6282 kb)

Additional file 2: **Table S2.** Sequence of primers used for qRT-PCR analysis. (XLS 7198 kb)

Additional file 3: **Dataset S1.** The 5-sample data set. Mean of the normalized expression value per transcript (FPKM fragments per kilobase of mapped reads) of the 33,191 transcripts at S2. (XLS 116 kb)

Additional file 4: **Figure S1.** Summary of the number of differentially expressed genes (DEGs) identified by RNA-seq analysis in the fruit skin tissues of ‘Red Delicious’ and its four generation mutants (XLS 17 kb)

Additional file 5: **Table S3.** Statistical table of the number of annotated differentially expressed genes (DEGs) (XLS 2153 kb)

Additional file 6: **Dataset S2.** Differentially expressed genes identified as commonly up-regulated or down-regulated in each pairwise comparison of ‘Red Delicious’ and its four generation mutants. (XLS 36 kb)

Additional file 7: **Dataset S3.** Gene composition of the six clusters identified using gene expression clustering analysis. (XLS 13 kb)

Additional file 8: **Table S4.** List of the 28 genes in phenylpropanoid biosynthesis and flavonoid biosynthesis pathways (ko00940 and ko00941) identified in ‘Red Delicious’ and its four generation mutants, their descriptions, loci, expression patterns and functional annotations. (XLS 12 kb)

Additional file 9: **Dataset S4.** Gene composition and mean FPKM value of the phenylpropanoid/flavonoid biosynthesis pathway. (TIF 392 kb)

Additional file 10: **Dataset S5.** Correlation analysis of anthocyanin content in S2 and MYB-like DNA-binding domain transcriptional factors. (DOC 359 kb)

Additional file 11: **Dataset S6.** Correlation analysis of anthocyanin content in S2 and helix-loop-helix DNA-binding domain transcriptional factors (DOC 29 kb)

Additional file 12: **Dataset S7.** Gene composition and mean FPKM value of the terpenoid biosynthesis pathway. The GO cellular component is reported. (DOC 34 kb)

Additional file 13: **Table S5.** List of the 18 genes in terpenoid backbone biosynthesis (ko00900) and steroid biosynthesis/sesquiterpenoid and triterpenoid biosynthesis pathways (ko00100/ko00909) identified in ‘Red Delicious’ and its four generation mutants, their descriptions, loci, expression patterns and functional annotations. (DOC 62 kb)

Additional file 14: **Dataset S8.** Gene composition and mean FPKM value of the plant hormone signal transduction. (DOC 49 kb)

Additional file 15: **Table S6.** List of the 12 genes in the plant hormone transduction pathway (ko04075) identified in ‘Red Delicious’ and its four generation mutants, their descriptions, locus, expression patterns and functional annotations. (DOC 35 kb)

## Abbreviations

4CL: 4-coumarate-CoA ligase
AACT1: Acetyltransferase, cytosolic 1
ANR: Anthocyanidin reductase
ANS: Anthocyanidin synthase
ASP3: Aspartate aminotransferase cytoplasmic
AUX1: Auxin; transporter-like 1
BGLU: Beta-glucosidase
CCoAOMT: Caffeoyl-CoA O-methyltransferase
CHI: Chalcone isomerase
CHS: Chalcone synthase
CYP98A: Cytochrome P450 98A2-like
DEGs: Differentially expressed genes
DFR: Dihydroflavonol reductase
F3’H: Flavonoid 3’-hydroxylase
FDR: False discovery rate
FLS: Flavonol synthase
FPPS2: Farnesyl pyrophosphate synthase 2-like
GO: Gene ontology
HCT: Quinate hydroxycinnamoyl transferase
HMGR: 3-hydroxy-3-methylglutaryl-coenzyme A reductase 1-like
HMGS: Hydroxymethylglutaryl-CoA synthase-like
HPLC: High-performance liquid chromatography
IDI1: Isopentenyl-diphosphate Delta-isomerase I
KEGG: Kyoto Encyclopedia of Genes and Genomes
LAR: Leucoanthocyanidin reductase
MVD2: Diphosphomevalonate decarboxylase MVD2-like
MVK: Mevalonate kinase-like
PAL: Phe ammonia lyase
PER: Peroxidases
PP2C: Protein phosphatase 2C
SnRK2: Sucrose non-fermenting-1-related protein kinase 2
SQMO: Squalene monooxygenase-like
