# Supplementary material for: Anthocyanin accumulation correlates with hormones in the fruit skin of ‘Red Delicious’ and its four generation bud sport mutants
Source: BMC Plant Biol. 2018 Dec 18;18:363. doi: 10.1186/s12870-018-1595-8 (PMC6299587; doi:10.1186/s12870-018-1595-8)
Supplement: Supplementary file 10 — Dataset S5. Correlation analysis of anthocyanin content in S2 and MYB-like DNA-binding domain transcriptional factors. (DOC 359 kb) [file 12870_2018_1595_MOESM10_ESM.doc]

**Supplemental Table S1: Gene Ontology (GO) enrichment analyses for DEGs in ‘Red Delicious’ and its four generation mutants.**

| **GO domain** | **GO. ID** | **Term** | **Annotated** | **Significant** | **Expected** | ***KS*<0.01** |
| --- | --- | --- | --- | --- | --- | --- |
| **Biological_process** |  | **G0 versus G1** |  |  |  |  |
|  | GO:0050832 | defense response to fungus | 72 | 3 | 1.11 | 0.0025 |
|  | GO:0007165 | signal transduction | 803 | 22 | 12.38 | 0.0025 |
|  | GO:0042218 | 1-aminocyclopropane-1-carboxylate biosynthetic process | 8 | 0 | 0.12 | 0.0026 |
|  | GO:0006032 | chitin catabolic process | 18 | 0 | 0.28 | 0.0044 |
|  | GO:0045926 | negative regulation of growth | 13 | 0 | 0.2 | 0.005 |
|  | GO:0009423 | chorismate biosynthetic process | 13 | 0 | 0.2 | 0.0066 |
|  | GO:2000024 | regulation of leaf development | 7 | 0 | 0.11 | 0.0067 |
|  | GO:0030244 | cellulose biosynthetic process | 70 | 1 | 1.08 | 0.0072 |
|  | GO:0045730 | respiratory burst | 10 | 1 | 0.15 | 0.0084 |
|  | GO:0071103 | DNA conformation change | 121 | 0 | 1.87 | 0.009 |
|  |  | **G0 versus G2** |  |  |  |  |
|  | GO:0050832 | defense response to fungus | 72 | 5 | 2.08 | 0.00083 |
|  | GO:0006032 | chitin catabolic process | 18 | 2 | 0.52 | 0.00117 |
|  | GO:0042218 | 1-aminocyclopropane-1-carboxylate biosynthetic process | 8 | 0 | 0.23 | 0.00321 |
|  | GO:0030244 | cellulose biosynthetic process | 70 | 3 | 2.02 | 0.00498 |
|  | GO:0010200 | response to chitin | 39 | 7 | 1.13 | 0.00617 |
|  | GO:0045926 | negative regulation of growth | 13 | 0 | 0.38 | 0.00653 |
|  | GO:2000024 | regulation of leaf development | 7 | 0 | 0.2 | 0.00762 |
|  | GO:0009423 | chorismate biosynthetic process | 13 | 0 | 0.38 | 0.00775 |
|  | GO:0009723 | response to ethylene | 59 | 7 | 1.7 | 0.00811 |
|  | GO:0009862 | systemic acquired resistance, salicylic acid mediated signaling pathway | 23 | 2 | 0.66 | 0.00962 |
|  | GO:0042742 | defense response to bacterium | 116 | 3 | 3.35 | 0.00964 |
|  | GO:0045730 | respiratory burst | 10 | 1 | 0.29 | 0.00995 |
|  |  | **G0 versus G3** |  |  |  |  |
|  | GO:0006355 | regulation of transcription, DNA-templated | 948 | 112 | 56.7 | 0.00075 |
|  | GO:1901700 | response to oxygen-containing compound | 526 | 53 | 31.46 | 0.00146 |
|  | GO:0045926 | negative regulation of growth | 13 | 1 | 0.78 | 0.00189 |
|  | GO:0009408 | response to heat | 98 | 19 | 5.86 | 0.00199 |
|  | GO:0001101 | response to acid chemical | 289 | 29 | 17.29 | 0.0023 |
|  | GO:0050832 | defense response to fungus | 72 | 9 | 4.31 | 0.00335 |
|  | GO:0006952 | defense response | 436 | 39 | 26.08 | 0.00466 |
|  | GO:0042218 | 1-aminocyclopropane-1-carboxylate biosynthetic process | 8 | 0 | 0.48 | 0.00524 |
|  | GO:0030243 | cellulose metabolic process | 83 | 2 | 4.96 | 0.00625 |
|  | GO:0071103 | DNA conformation change | 121 | 7 | 7.24 | 0.00724 |
|  | GO:0007165 | signal transduction | 803 | 57 | 48.03 | 0.00878 |
|  | GO:0012501 | programmed cell death | 73 | 7 | 4.37 | 0.00991 |
|  |  | **G0 versus G4** |  |  |  |  |
|  | GO:0055114 | oxidation-reduction process | 1696 | 138 | 112.37 | 0.001 |
|  | GO:0009765 | photosynthesis, light harvesting | 35 | 12 | 2.32 | 0.0017 |
|  | GO:0001101 | response to acid chemical | 289 | 30 | 19.15 | 0.0019 |
|  | GO:1901700 | response to oxygen-containing compound | 526 | 59 | 34.85 | 0.0021 |
|  | GO:0045926 | negative regulation of growth | 13 | 2 | 0.86 | 0.0022 |
|  | GO:0006801 | superoxide metabolic process | 22 | 6 | 1.46 | 0.0028 |
|  | GO:0052545 | callose localization | 15 | 5 | 0.99 | 0.0048 |
|  | GO:0006952 | defense response | 436 | 37 | 28.89 | 0.0058 |
|  | GO:0042218 | 1-aminocyclopropane-1-carboxylate biosynthetic process | 8 | 0 | 0.53 | 0.0059 |
|  | GO:0010200 | response to chitin | 39 | 8 | 2.58 | 0.0062 |
|  | GO:0009642 | response to light intensity | 84 | 13 | 5.57 | 0.0066 |
|  | GO:0030243 | cellulose metabolic process | 83 | 2 | 5.5 | 0.0081 |
|  | GO:0031408 | oxylipin biosynthetic process | 31 | 4 | 2.05 | 0.0087 |
|  | GO:0051274 | beta-glucan biosynthetic process | 74 | 4 | 4.9 | 0.0088 |
|  | GO:0043650 | dicarboxylic acid biosynthetic process | 17 | 1 | 1.13 | 0.0092 |
|  | GO:0051716 | cellular response to stimulus | 1095 | 73 | 72.55 | 0.0099 |
|  | GO:0050832 | defense response to fungus | 72 | 6 | 4.77 | 0.0099 |
|  |  | **G1 versus G2** |  |  |  |  |
|  | GO:0006032 | chitin catabolic process | 18 | 1 | 0.06 | 0.00062 |
|  | GO:0030243 | cellulose metabolic process | 83 | 0 | 0.29 | 0.00177 |
|  | GO:0042218 | 1-aminocyclopropane-1-carboxylate biosynthetic process | 8 | 0 | 0.03 | 0.00209 |
|  | GO:0045926 | negative regulation of growth | 13 | 0 | 0.05 | 0.00393 |
|  | GO:0006952 | defense response | 436 | 2 | 1.51 | 0.00444 |
|  | GO:0009423 | chorismate biosynthetic process | 13 | 0 | 0.05 | 0.00542 |
|  | GO:2000024 | regulation of leaf development | 7 | 0 | 0.02 | 0.00586 |
|  | GO:0071103 | DNA conformation change | 121 | 0 | 0.42 | 0.00679 |
|  |  | **G2 versus G3** |  |  |  |  |
|  | GO:0006032 | chitin catabolic process | 18 | 3 | 0.31 | 0.00089 |
|  | GO:0006952 | defense response | 436 | 13 | 7.61 | 0.00115 |
|  | GO:0042218 | 1-aminocyclopropane-1-carboxylate biosynthetic process | 8 | 0 | 0.14 | 0.00267 |
|  | GO:0030243 | cellulose metabolic process | 83 | 0 | 1.45 | 0.00311 |
|  | GO:0045926 | negative regulation of growth | 13 | 0 | 0.23 | 0.00524 |
|  | GO:0007165 | signal transduction | 803 | 22 | 14.01 | 0.00612 |
|  | GO:0009423 | chorismate biosynthetic process | 13 | 0 | 0.23 | 0.0067 |
|  | GO:2000024 | regulation of leaf development | 7 | 0 | 0.12 | 0.00684 |
|  | GO:0006972 | hyperosmotic response | 64 | 1 | 1.12 | 0.00716 |
|  | GO:0048508 | embryonic meristem development | 15 | 1 | 0.26 | 0.00802 |
|  | GO:0007568 | aging | 40 | 1 | 0.7 | 0.00933 |
|  | GO:0071103 | DNA conformation change | 121 | 0 | 2.11 | 0.00935 |
|  |  | **G3 versus G4** |  |  |  |  |
|  | GO:0042218 | 1-aminocyclopropane-1-carboxylate biosynthetic process | 8 | 0 | 0.08 | 0.0024 |
|  | GO:0030243 | cellulose metabolic process | 83 | 0 | 0.87 | 0.0024 |
|  | GO:0006032 | chitin catabolic process | 18 | 0 | 0.19 | 0.004 |
|  | GO:0045926 | negative regulation of growth | 13 | 0 | 0.14 | 0.0046 |
|  | GO:0009423 | chorismate biosynthetic process | 13 | 0 | 0.14 | 0.0061 |
|  | GO:2000024 | regulation of leaf development | 7 | 0 | 0.07 | 0.0064 |
|  | GO:0071103 | DNA conformation change | 121 | 0 | 1.27 | 0.0084 |
|  | GO:0007568 | aging | 40 | 2 | 0.42 | 0.0085 |
|  | GO:0043562 | cellular response to nitrogen levels | 6 | 1 | 0.06 | 0.0096 |
|  | GO:0050832 | defense response to fungus | 72 | 1 | 0.76 | 0.0097 |
|  |  |  |  |  |  |  |
| **Cellular Component** |  | **G0 versus G1** |  |  |  |  |
|  | GO:0033588 | Elongator holoenzyme complex | 7 | 0 | 0.1 | 0.0059 |
|  | GO:0005758 | mitochondrial intermembrane space | 13 | 0 | 0.19 | 0.0086 |
|  | GO:0005689 | U12-type spliceosomal complex | 12 | 0 | 0.18 | 0.0097 |
|  | GO:0005694 | chromosome | 142 | 0 | 2.13 | 0.01 |
|  |  | **G0 versus G2** |  |  |  |  |
|  | GO:0033588 | Elongator holoenzyme complex | 7 | 0 | 0.19 | 0.0067 |
|  | GO:0048046 | apoplast | 174 | 15 | 4.7 | 0.0098 |
|  |  | **G0 versus G3** |  |  |  |  |
|  | GO:0005576 | extracellular region | 337 | 43 | 18.78 | 0.0024 |
|  | GO:0005758 | mitochondrial intermembrane space | 13 | 1 | 0.72 | 0.0031 |
|  | GO:0000786 | nucleosome | 82 | 7 | 4.57 | 0.0034 |
|  | GO:0033588 | Elongator holoenzyme complex | 7 | 0 | 0.39 | 0.009 |
|  |  | **G0 versus G4** |  |  |  |  |
|  | GO:0005758 | mitochondrial intermembrane space | 13 | 2 | 0.81 | 0.0037 |
|  | GO:0048046 | apoplast | 174 | 23 | 10.89 | 0.0071 |
|  | GO:0005618 | cell wall | 262 | 32 | 16.4 | 0.0085 |
|  | GO:0033588 | Elongator holoenzyme complex | 7 | 0 | 0.44 | 0.0097 |
|  |  | **G1 versus G2** |  |  |  |  |
|  | GO:0005694 | chromosome | 142 | 0 | 0.38 | 0.0045 |
|  | GO:0033588 | Elongator holoenzyme complex | 7 | 0 | 0.02 | 0.005 |
|  | GO:0005758 | mitochondrial intermembrane space | 13 | 0 | 0.04 | 0.0067 |
|  | GO:0005689 | U12-type spliceosomal complex | 12 | 0 | 0.03 | 0.0077 |
|  |  | **G2 versus G3** |  |  |  |  |
|  | GO:0005758 | mitochondrial intermembrane space | 13 | 1 | 0.22 | 0.0014 |
|  | GO:0033588 | Elongator holoenzyme complex | 7 | 0 | 0.12 | 0.0059 |
|  | GO:0005689 | U12-type spliceosomal complex | 12 | 0 | 0.21 | 0.01 |
|  |  | **G3 versus G4** |  |  |  |  |
|  | GO:0033588 | Elongator holoenzyme complex | 7 | 0 | 0.07 | 0.0054 |
|  | GO:0005694 | chromosome | 142 | 0 | 1.32 | 0.0068 |
|  | GO:0005758 | mitochondrial intermembrane space | 13 | 0 | 0.12 | 0.0076 |
|  | GO:0005689 | U12-type spliceosomal complex | 12 | 0 | 0.11 | 0.0087 |
| **Molecular_function** |  | **G0 versus G1** |  |  |  |  |
|  | GO:0043531 | ADP binding | 77 | 2 | 1.25 | 1.70E-10 |
|  | GO:0003939 | L-iditol 2-dehydrogenase activity | 18 | 0 | 0.29 | 6.70E-07 |
|  | GO:0047617 | acyl-CoA hydrolase activity | 6 | 0 | 0.1 | 2.90E-05 |
|  | GO:0003854 | 3-beta-hydroxy-delta5-steroid dehydrogenase activity | 15 | 0 | 0.24 | 5.00E-05 |
|  | GO:0008171 | O-methyltransferase activity | 48 | 0 | 0.78 | 0.00028 |
|  | GO:0016847 | 1-aminocyclopropane-1-carboxylate synthase activity | 8 | 0 | 0.13 | 0.00256 |
|  | GO:0016210 | naringenin-chalcone synthase activity | 7 | 0 | 0.11 | 0.00293 |
|  | GO:0005488 | binding | 7301 | 137 | 118.29 | 0.0033 |
|  | GO:0004097 | catechol oxidase activity | 16 | 0 | 0.26 | 0.00365 |
|  | GO:0016710 | trans-cinnamate 4-monooxygenase activity | 6 | 0 | 0.1 | 0.00498 |
|  | GO:0004620 | phospholipase activity | 32 | 2 | 0.52 | 0.00619 |
|  | GO:0004568 | chitinase activity | 19 | 0 | 0.31 | 0.00757 |
|  | GO:0046982 | protein heterodimerization activity | 96 | 1 | 1.56 | 0.00916 |
|  | GO:1901681 | sulfur compound binding | 28 | 0 | 0.45 | 0.00917 |
|  | GO:0004857 | enzyme inhibitor activity | 65 | 2 | 1.05 | 0.00948 |
|  | GO:0047763 | caffeate O-methyltransferase activity | 13 | 0 | 0.21 | 0.00966 |
|  |  | **G0 versus G2** |  |  |  |  |
|  | GO:0043531 | ADP binding | 77 | 6 | 2.36 | 4.40E-10 |
|  | GO:0003939 | L-iditol 2-dehydrogenase activity | 18 | 0 | 0.55 | 1.20E-06 |
|  | GO:0008171 | O-methyltransferase activity | 48 | 1 | 1.47 | 1.50E-05 |
|  | GO:0047617 | acyl-CoA hydrolase activity | 6 | 0 | 0.18 | 4.00E-05 |
|  | GO:0016210 | naringenin-chalcone synthase activity | 7 | 4 | 0.21 | 4.90E-05 |
|  | GO:0003854 | 3-beta-hydroxy-delta5-steroid dehydrogenase activity | 15 | 1 | 0.46 | 6.60E-05 |
|  | GO:0008061 | chitin binding | 6 | 2 | 0.18 | 0.00084 |
|  | GO:0004568 | chitinase activity | 19 | 2 | 0.58 | 0.00231 |
|  | GO:0045430 | chalcone isomerase activity | 9 | 1 | 0.28 | 0.00325 |
|  | GO:0016847 | 1-aminocyclopropane-1-carboxylate synthase activity | 8 | 0 | 0.25 | 0.00327 |
|  | GO:0004097 | catechol oxidase activity | 16 | 1 | 0.49 | 0.00416 |
|  | GO:0016759 | cellulose synthase activity | 24 | 2 | 0.74 | 0.00452 |
|  | GO:0020037 | heme binding | 278 | 14 | 8.54 | 0.00508 |
|  | GO:0016630 | protochlorophyllide reductase activity | 13 | 3 | 0.4 | 0.00523 |
|  | GO:0016710 | trans-cinnamate 4-monooxygenase activity | 6 | 0 | 0.18 | 0.00603 |
|  | GO:0046943 | carboxylic acid transmembrane transporter activity | 38 | 2 | 1.17 | 0.00626 |
|  | GO:0043169 | cation binding | 2372 | 89 | 72.83 | 0.0075 |
|  | GO:0004620 | phospholipase activity | 32 | 2 | 0.98 | 0.00796 |
|  |  | **G0 versus G3** |  |  |  |  |
|  | GO:0043531 | ADP binding | 77 | 5 | 4.58 | 8.40E-10 |
|  | GO:0003939 | L-iditol 2-dehydrogenase activity | 18 | 1 | 1.07 | 3.90E-06 |
|  | GO:0008171 | O-methyltransferase activity | 48 | 3 | 2.86 | 4.10E-05 |
|  | GO:0047617 | acyl-CoA hydrolase activity | 6 | 0 | 0.36 | 7.30E-05 |
|  | GO:0016210 | naringenin-chalcone synthase activity | 7 | 4 | 0.42 | 8.60E-05 |
|  | GO:0003854 | 3-beta-hydroxy-delta5-steroid dehydrogenase activity | 15 | 1 | 0.89 | 0.00012 |
|  | GO:0003700 | sequence-specific DNA binding transcription factor activity | 445 | 68 | 26.5 | 0.00021 |
|  | GO:0003677 | DNA binding | 1125 | 114 | 66.99 | 0.00108 |
|  | GO:0046872 | metal ion binding | 2330 | 167 | 138.74 | 0.00306 |
|  | GO:0005516 | calmodulin binding | 40 | 6 | 2.38 | 0.00431 |
|  | GO:0045430 | chalcone isomerase activity | 9 | 4 | 0.54 | 0.00463 |
|  | GO:0016847 | 1-aminocyclopropane-1-carboxylate synthase activity | 8 | 0 | 0.48 | 0.00519 |
|  | GO:0004097 | catechol oxidase activity | 16 | 1 | 0.95 | 0.00567 |
|  | GO:0047641 | aldose-6-phosphate reductase (NADPH) activity | 6 | 2 | 0.36 | 0.00677 |
|  | GO:0046906 | tetrapyrrole binding | 297 | 20 | 17.68 | 0.00819 |
|  | GO:1901681 | sulfur compound binding | 28 | 3 | 1.67 | 0.00841 |
|  | GO:0016710 | trans-cinnamate 4-monooxygenase activity | 6 | 0 | 0.36 | 0.00873 |
|  | GO:0043169 | cation binding | 2372 | 171 | 141.24 | 0.00991 |
|  |  | **G0 versus G4** |  |  |  |  |
|  | GO:0043531 | ADP binding | 77 | 5 | 5.17 | 1.50E-09 |
|  | GO:0003939 | L-iditol 2-dehydrogenase activity | 18 | 2 | 1.21 | 4.70E-07 |
|  | GO:0008171 | O-methyltransferase activity | 48 | 3 | 3.22 | 5.40E-05 |
|  | GO:0047617 | acyl-CoA hydrolase activity | 6 | 0 | 0.4 | 8.60E-05 |
|  | GO:0016210 | naringenin-chalcone synthase activity | 7 | 4 | 0.47 | 0.0001 |
|  | GO:0003854 | 3-beta-hydroxy-delta5-steroid dehydrogenase activity | 15 | 2 | 1.01 | 0.00014 |
|  | GO:0020037 | heme binding | 278 | 27 | 18.67 | 0.0028 |
|  | GO:0046872 | metal ion binding | 2330 | 184 | 156.48 | 0.00299 |
|  | GO:0003700 | sequence-specific DNA binding transcription factor activity | 445 | 58 | 29.89 | 0.00459 |
|  | GO:0004620 | phospholipase activity | 32 | 4 | 2.15 | 0.00538 |
|  | GO:0005516 | calmodulin binding | 40 | 7 | 2.69 | 0.00571 |
|  | GO:0016847 | 1-aminocyclopropane-1-carboxylate synthase activity | 8 | 0 | 0.54 | 0.00591 |
|  | GO:0004097 | catechol oxidase activity | 16 | 2 | 1.07 | 0.00631 |
|  | GO:0047641 | aldose-6-phosphate reductase (NADPH) activity | 6 | 2 | 0.4 | 0.00753 |
|  | GO:0016710 | trans-cinnamate 4-monooxygenase activity | 6 | 0 | 0.4 | 0.0097 |
|  | GO:0016840 | carbon-nitrogen lyase activity | 20 | 4 | 1.34 | 0.0099 |
|  | GO:0047668 | amygdalin beta-glucosidase activity | 12 | 1 | 0.81 | 0.00992 |
|  |  | **G1 versus G2** |  |  |  |  |
|  | GO:0043531 | ADP binding | 77 | 1 | 0.23 | 5.60E-11 |
|  | GO:0003939 | L-iditol 2-dehydrogenase activity | 18 | 0 | 0.05 | 3.80E-07 |
|  | GO:0047617 | acyl-CoA hydrolase activity | 6 | 0 | 0.02 | 2.20E-05 |
|  | GO:0003854 | 3-beta-hydroxy-delta5-steroid dehydrogenase activity | 15 | 0 | 0.04 | 3.80E-05 |
|  | GO:0008171 | O-methyltransferase activity | 48 | 1 | 0.14 | 4.00E-05 |
|  | GO:0008061 | chitin binding | 6 | 1 | 0.02 | 0.00056 |
|  | GO:0004568 | chitinase activity | 19 | 1 | 0.06 | 0.00115 |
|  | GO:0016847 | 1-aminocyclopropane-1-carboxylate synthase activity | 8 | 0 | 0.02 | 0.00203 |
|  | GO:0016210 | naringenin-chalcone synthase activity | 7 | 0 | 0.02 | 0.0024 |
|  | GO:0004097 | catechol oxidase activity | 16 | 0 | 0.05 | 0.00308 |
|  | GO:0016710 | trans-cinnamate 4-monooxygenase activity | 6 | 0 | 0.02 | 0.00413 |
|  | GO:0020037 | heme binding | 278 | 6 | 0.83 | 0.00426 |
|  | GO:1901681 | sulfur compound binding | 28 | 1 | 0.08 | 0.00646 |
|  | GO:0047763 | caffeate O-methyltransferase activity | 13 | 0 | 0.04 | 0.008 |
|  | GO:0016746 | transferase activity, transferring acyl groups | 293 | 2 | 0.88 | 0.00818 |
|  |  | **G2 versus G3** |  |  |  |  |
|  | GO:0043531 | ADP binding | 77 | 2 | 1.33 | 1.70E-10 |
|  | GO:0003939 | L-iditol 2-dehydrogenase activity | 18 | 0 | 0.31 | 7.10E-07 |
|  | GO:0047617 | acyl-CoA hydrolase activity | 6 | 0 | 0.1 | 3.00E-05 |
|  | GO:0003854 | 3-beta-hydroxy-delta5-steroid dehydrogenase activity | 15 | 0 | 0.26 | 5.10E-05 |
|  | GO:0008171 | O-methyltransferase activity | 48 | 2 | 0.83 | 7.80E-05 |
|  | GO:0008061 | chitin binding | 6 | 3 | 0.1 | 0.0007 |
|  | GO:0004568 | chitinase activity | 19 | 3 | 0.33 | 0.0017 |
|  | GO:0016847 | 1-aminocyclopropane-1-carboxylate synthase activity | 8 | 0 | 0.14 | 0.0026 |
|  | GO:0016210 | naringenin-chalcone synthase activity | 7 | 0 | 0.12 | 0.003 |
|  | GO:0004097 | catechol oxidase activity | 16 | 0 | 0.28 | 0.0036 |
|  | GO:0020037 | heme binding | 278 | 12 | 4.82 | 0.0046 |
|  | GO:0016710 | trans-cinnamate 4-monooxygenase activity | 6 | 0 | 0.1 | 0.005 |
|  | GO:0046872 | metal ion binding | 2330 | 61 | 40.39 | 0.0075 |
|  | GO:1901681 | sulfur compound binding | 28 | 2 | 0.49 | 0.0094 |
|  | GO:0008514 | organic anion transmembrane transporter activity | 78 | 2 | 1.35 | 0.0096 |
|  | GO:0047763 | caffeate O-methyltransferase activity | 13 | 0 | 0.23 | 0.0098 |
|  |  | **G3 versus G4** |  |  |  |  |
|  | GO:0043531 | ADP binding | 77 | 1 | 0.87 | 1.10E-10 |
|  | GO:0003939 | L-iditol 2-dehydrogenase activity | 18 | 1 | 0.2 | 3.80E-08 |
|  | GO:0047617 | acyl-CoA hydrolase activity | 6 | 0 | 0.07 | 2.60E-05 |
|  | GO:0003854 | 3-beta-hydroxy-delta5-steroid dehydrogenase activity | 15 | 0 | 0.17 | 4.50E-05 |
|  | GO:0008171 | O-methyltransferase activity | 48 | 1 | 0.54 | 0.00024 |
|  | GO:0016847 | 1-aminocyclopropane-1-carboxylate synthase activity | 8 | 0 | 0.09 | 0.00235 |
|  | GO:0016210 | naringenin-chalcone synthase activity | 7 | 0 | 0.08 | 0.00273 |
|  | GO:0004097 | catechol oxidase activity | 16 | 0 | 0.18 | 0.00344 |
|  | GO:0008514 | organic anion transmembrane transporter activity | 78 | 2 | 0.88 | 0.00375 |
|  | GO:0016710 | trans-cinnamate 4-monooxygenase activity | 6 | 0 | 0.07 | 0.00464 |
|  | GO:0004568 | chitinase activity | 19 | 0 | 0.22 | 0.00682 |
|  | GO:0046906 | tetrapyrrole binding | 297 | 5 | 3.37 | 0.00695 |
|  | GO:1901681 | sulfur compound binding | 28 | 1 | 0.32 | 0.00819 |
|  | GO:0047763 | caffeate O-methyltransferase activity | 13 | 0 | 0.15 | 0.00899 |
