# Supplementary material for: Anthocyanin accumulation correlates with hormones in the fruit skin of ‘Red Delicious’ and its four generation bud sport mutants
Source: BMC Plant Biol. 2018 Dec 18;18:363. doi: 10.1186/s12870-018-1595-8 (PMC6299587; doi:10.1186/s12870-018-1595-8)
Supplement: Supplementary file 11 — Dataset S6. Correlation analysis of anthocyanin content in S2 and helix-loop-helix DNA-binding domain transcriptional factors (DOC 29 kb) [file 12870_2018_1595_MOESM11_ESM.doc]

**Supplemental Table S2:** Sequence of primers used for qRT-PCR analysis.

| **Gene_ID** | **Gene name** | **Forward primer (5' to 3')** | **Reverse primer (5' to 3')** |
| --- | --- | --- | --- |
| MD00G1088100 | CCoAOMT | TCCACTCCGCAAGTACGTCAGG | TGATGCCATCACCAACAGGAAGC |
| MD01G1162400 | *PER* | GCTGCCAACACCTCCATTCCTC | CGGAGTCGTTGTAGATGCGTGAG |
| MD01G1236300 | *4CL* | GGAACCGATGCCAACCAAGTCAG | TGCCGTAGCCGCAACATCATTC |
| MD04G1096200 | *PAL1* | ACCAGAACGGAGTGTCGGAGTC | TGAGTGGCTATGGCTGCAACTTG |
| MD05G1312800 | *BGLU* | GGTGACACACCACCAACTCC | TTTGCCTTCGAAGTGGGCTT |
| MD08G1117400 | *ASP3* | GGCAGGTACTCCTGGTGACT | GGCAACTTGTTCAGGGCTCA |
| MD04G1003300 | *CHS* | TCGGACTGGAACTCACTCTTCTGG | GGCGGACTTCCTCCTGACCTC |
| MD06G1201700 | *F3'H* | TCGGACCTGCCTAACTTGACCTAC | GCAACTCTCGGACGCCATTCG |
| MD08G1243000 | *CYP98A3* | CTGACAGGCACAGGAACAGATCG | GCACGCCTTCCTCACCTTCAC |
| MD13G1285100 | *CHS* | TCGGACTGGAACTCACTCTTCTGG | TGCCGTAATCTGACAACACTTGCC |
| MD02G1132200 | *F3H* | AAGGTTGCCTACAACGACTTCAGC | CAGTCCTCACAAGCCGCTACAATC |
| MD14G1155800 | *HCT* | AAGTGTGCAAGCTCAACCTCTGG | CACGAAGCCAGGCAACTCTATCTC |
| MD09G1128400 | *AACT1* | TTGTTGTGGCTGGTGGCATGG | CCACAGACCATCCTTCAGCATTCC |
| MD00G1033700 | *SAUR* | CGTGATTGCGGTGGATGGAGAC | TTGGCTGGCAAGGAATGGTTAGG |
| MD15G1075800 | *GID2* | ACAAGACGGCGGAGGACGAG | GAGCTGGAAGAGGCGGAGGAG |
| MD07G1279300 | *RVE6* | GCTGAACAACAGTTGCAGTACC | AGACGCTGCCAATGAATCCG |
| EB146750 | *GADPH* | TGAGGGCAAGGTGAAGGGTATCTT | TCAAGTCAACCACACGCGTACTGT |
