# Supplementary material for: Anthocyanin accumulation correlates with hormones in the fruit skin of ‘Red Delicious’ and its four generation bud sport mutants
Source: BMC Plant Biol. 2018 Dec 18;18:363. doi: 10.1186/s12870-018-1595-8 (PMC6299587; doi:10.1186/s12870-018-1595-8)
Supplement: Supplementary file 12 — Dataset S7. Gene composition and mean FPKM value of the terpenoid biosynthesis pathway. The GO cellular component is reported. (DOC 34 kb) [file 12870_2018_1595_MOESM12_ESM.doc]

**Supplemental Table S3: Statistical table of the number of annotated differentially expressed genes (DEGs).**

| **DEG Set** | **Total** | **COG** | **GO** | **KEGG** | **KOG** | **NR** | **Pfam** | **Swiss-Prot** | **eggNOG** |
| --- | --- | --- | --- | --- | --- | --- | --- | --- | --- |
| G0 vs. G1 | 628 | 0 | 250 | 168 | 288 | 626 | 547 | 503 | 8 |
| G0 vs. G2 | 1,175 | 1 | 448 | 358 | 556 | 1,174 | 1,003 | 926 | 11 |
| G0 vs. G3 | 2,234 | 2 | 891 | 682 | 1,092 | 2,232 | 1,926 | 1,816 | 18 |
| G0 vs. G4 | 2,517 | 3 | 994 | 768 | 1,222 | 2,514 | 2,175 | 2,036 | 22 |
| G1 vs. G2 | 113 | 1 | 50 | 45 | 53 | 113 | 95 | 91 | 1 |
| G2 vs. G3 | 678 | 2 | 273 | 214 | 312 | 677 | 599 | 539 | 7 |
| G3 vs. G4 | 392 | 2 | 168 | 115 | 183 | 391 | 333 | 311 | 8 |
