# Supplementary material for: Anthocyanin accumulation correlates with hormones in the fruit skin of ‘Red Delicious’ and its four generation bud sport mutants
Source: BMC Plant Biol. 2018 Dec 18;18:363. doi: 10.1186/s12870-018-1595-8 (PMC6299587; doi:10.1186/s12870-018-1595-8)
Supplement: Supplementary file 13 — Table S5. List of the 18 genes in terpenoid backbone biosynthesis (ko00900) and steroid biosynthesis/sesquiterpenoid and triterpenoid biosynthesis pathways (ko00100/ko00909) identified in ‘Red Delicious’ and its four generation mutants, their descriptions, loci, expression patterns and functional annotations. (DOC 62 kb) [file 12870_2018_1595_MOESM13_ESM.doc]

**Supplemental** **Table S4:** List of the 28 genes in phenylpropanoid biosynthesis and flavonoid biosynthesis pathway (ko00940 and ko00941) were identified in ‘Red Delicious’ and its four generation mutants, their descriptions, locus, expression patterns and functional annotations.

| **Gene_ID** | **Gene description** | **Locus** | **Strand** | **Expression pattern** |
| --- | --- | --- | --- | --- |
| MD00G1088100 | caffeoyl-CoA O-methyltransferase (CCoAOMT) | Chr00:17871472-17873066 | - | Cluster 1 |
| MD01G1118300 | chalcone--flavonone isomerase (CHI) | Chr01:23211441-23212288 | - |
| MD08G1168600 | flavonol synthase (FLS) | Chr08:20082535-20087388 | + |
| MD09G1226600 | quinate hydroxycinnamoyl transferase (*HCT*) | Chr09:28007490-28011281 | + |
| MD01G1162400 | peroxidase 4-like (*PER4*) | Chr01:26724247-26725944 | - | Cluster 2 |
| MD01G1236300 | 4-coumarate--CoA ligase 2-like (*4CL*) | Chr01:32373035-32379246 | - |
| MD04G1003300 | chalcone synthase (*CHS*) | Chr04:488057-489737 | - |
| MD04G1096200 | Phe ammonia lyase 1 (*PAL1*) | Chr04:17723731-17727188 | - |
| MD05G1312800 | beta-glucosidase 24 (*BGLU24*) | Chr05:44347183-44358866 | + |
| MD05G1345800 | peroxidase 42 (*PER42*) | Chr05:46533897-46536242 | - |
| MD06G1201700 | flavonoid 3' hydroxylase (*F3'H*) | Chr06:33501191-33504591 | + |
| MD06G1229100 | beta-glucosidase 44 (*BGLU44*) | Chr06:35898325-35902567 | - |
| MD08G1117400 | aspartate aminotransferase, cytoplasmic-like (*ASP3*) | Chr08:10736594-10737144 | + |
| MD08G1243000 | cytochrome P450 98A2-like (*CYP98A3*) | Chr08:30847344-30850077 | - |
| MD13G1246700 | Peroxidase 16 (*PER16*) | Chr13:25989270-25991264 | + |
| MD13G1285100 | chalcone synthase (*CHS*) | Chr13:43203447-43206049 | + |
| MD14G1210700 | flavanone 3' hydroxylase (*F3'H*) | Chr14:29630776-29634806 | + |
| MD02G1132200 | flavanone 3-hydroxylase (*F3H*) | Chr02:10684585-10686985 | - | Cluster 3 |
| MD03G1001100 | anthocyanidin synthase (*ANS*) | Chr03:67749-70084 | + |
| MD04G1003000 | chalcone synthase (*CHS*) | Chr04:435282-440567 | + |
| MD04G1003400 | chalcone synthase (*CHS*) | Chr04:500950-503483 | + |
| MD06G1071600 | anthocyanidin synthase (*ANS*) | Chr06:17544096-17545587 | + |
| MD07G1186300 | chalcone--flavonone isomerase (CHI) | Chr07:26733159-26735913 | - |
| MD08G1121600 | flavonol synthase (FLS) | Chr08:11151868-11158461 | + |
| MD08G1242900 | cytochrome P450 98A2-like (*CYP98A3*) | Chr08:30838332-30842953 | - |
| MD15G1024100 | dihydroflavonol reductase (*DFR*) | Chr15:1433884-1436591 | + |
| MD15G1436500 | cytochrome P450 98A2-like (*CYP98A3*) | Chr15:53651165-53655331 | - |
| MD14G1155800 | shikimate O-hydroxycinnamoyltransferase (*HCT*) | Chr14:24997548-24998822 | - | Cluster 5 |
