# Supplementary material for: Anthocyanin accumulation correlates with hormones in the fruit skin of ‘Red Delicious’ and its four generation bud sport mutants
Source: BMC Plant Biol. 2018 Dec 18;18:363. doi: 10.1186/s12870-018-1595-8 (PMC6299587; doi:10.1186/s12870-018-1595-8)
Supplement: Supplementary file 14 — Dataset S8. Gene composition and mean FPKM value of the plant hormone signal transduction. (DOC 49 kb) [file 12870_2018_1595_MOESM14_ESM.doc]

**Supplemental Table S5:** List of the 18 genes in terpenoid backbone biosynthesis (ko00900) and steroid biosynthesis / sesquiterpenoid and triterpenoid biosynthesis pathway (ko00100 / ko00909) were identified in ‘Red Delicious’ and its four generation mutants, their descriptions, locus, expression patterns and functional annotations.

| **Gene_ID** | **Gene description** | **Locus** | **Strand** | **Expression pattern** |
| --- | --- | --- | --- | --- |
| MD02G1256300 | diphosphomevalonate decarboxylase MVD2-like (*MVD2*) | Chr02:30854526-30859747 | - | Cluster 1 |
| MD04G1069200 | hydroxymethylglutaryl-CoA synthase-like (*HMGS*) | Chr04:9471788-9476385 | + |
| MD04G1245100 | isopentenyl-diphosphate Delta-isomerase I (*IDI1*) | Chr04:32086167-32088962 | - |
| MD05G1143500 | 3-hydroxy-3-methylglutaryl-coenzyme A reductase 1-like (*HMGR*) | Chr05:27469980-27472553 | + |
| MD05G1362500 | farnesyl pyrophosphate synthase 2-like (*FPPS2*) | Chr05:47762058-47766099 | + |
| MD06G1070000 | hydroxymethylglutaryl-CoA synthase-like (*HMGS*) | Chr06:16956857-16961198 | + |
| MD07G1064100 | diphosphomevalonate decarboxylase MVD2-like (*MVD2*) | Chr07: 6017376-6022701 | + |
| MD09G1128400 | acetyl-CoA acetyltransferase, cytosolic 1 (*AACT1*) | Chr09:9927093-9931596 | + |
| MD10G1340100 | farnesyl pyrophosphate synthase 2 (*FPPS2*) | Chr10:41560466-41564657 | + |
| MD11G1036800 | farnesyl pyrophosphate synthase 2-like (*FPPS2*) | Chr11:3180305 3184669 | + |
| MD12G1220700 | mevalonate kinase-like (*MVK*) | Chr12:29732977-29737145 | - |
| MD16G1201100 | 3-hydroxy-3-methylglutaryl-coenzyme A reductase 1-like (*HMGR*) | Chr16:18188074-18188979 | + |
| MD05G1112000 | squalene monooxygenase-like (*SQMO*) | Chr05:22908492-22911688 | - | Cluster 4 |
| MD05G1112100 | squalene monooxygenase-like (*SQMO*) | Chr05:22970009-22973213 | - |
| MD05G1112200 | squalene monooxygenase-like (*SQMO*) | Chr05:23066012-23069192 | - |
| MD08G1076800 | squalene monooxygenase-like (*SQMO*) | Chr08:6291368-6295808 | - |
| MD07G1041500 | squalene monooxygenase-like (*SQMO*) | Chr07:3454916-3457994 | - | Cluster 5 |
| MD07G1041600 | squalene monooxygenase-like (*SQMO*) | Chr07:3465051-3468134 | + |
