# Supplementary material for: Anthocyanin accumulation correlates with hormones in the fruit skin of ‘Red Delicious’ and its four generation bud sport mutants
Source: BMC Plant Biol. 2018 Dec 18;18:363. doi: 10.1186/s12870-018-1595-8 (PMC6299587; doi:10.1186/s12870-018-1595-8)
Supplement: Supplementary file 15 — Table S6. List of the 12 genes in the plant hormone transduction pathway (ko04075) identified in ‘Red Delicious’ and its four generation mutants, their descriptions, locus, expression patterns and functional annotations. (DOC 35 kb) [file 12870_2018_1595_MOESM15_ESM.doc]

**Supplemental Table S6:** List of the 12 genes in plant hormone transduction pathway (ko04075) were identified in ‘Red Delicious’ and its four generation mutants, their descriptions, locus, expression patterns and functional annotations.

| **Gene_ID** | **Gene description** | **Locus** | **Strand** | **Expression pattern** |
| --- | --- | --- | --- | --- |
| MD00G1033700 | auxin-responsive protein SAUR50 (*SAUR50*) | Chr00:5801113-5801541 | - | Cluster 2 |
| MD01G1149100 | auxin transporter-like protein 1 (*AUX1*) | Chr01:25776734-25780967 | + |
| MD02G1057200 | auxin-responsive protein IAA27 (*IAA27*) | Chr02:4631406-4634380 | + |
| MD06G1046300 | serine/threonine-protein kinase SAPK3-like (*SAPK3*) / sucrose non-fermenting-1-related protein kinase 2.4 (*SnRK2.4*) | Chr06:6293617-6297175 | + |
| MD07G1215900 | auxin transporter-like protein 2 (*AUX1*) | Chr07:29315572-29319808 | + |
| MD09G1202300 | auxin-responsive protein IAA26-like (*IAA26*) | Chr09:18823904-18826992 | - |
| MD10G1061300 | auxin-responsive protein SAUR50 (*SAUR50*) | Chr10:8240210-8240869 | + |
| MD10G1192900 | auxin-induced protein AUX28 (*AUX28*) | Chr10:28909337-28912808 | - |
| MD15G1014200 | auxin-responsive protein SAUR50 (*SAUR50*) | Chr15:805247-805606 | + |
| MD15G1075800 | F-box protein GID2-like (*GID2*) | Chr15:5172735-5173331 | + |
| MD15G1191800 | auxin-responsive protein IAA27-like (*IAA27*) | Chr15:15104026-15107317 | + |
| MD15G1195800 | protein phosphatase 2C (*PP2C*) | Chr15:15554449-15558703 | + |
